# Glycyrrhizin Derivatives Suppress Cancer Chemoresistance by Inhibiting Progesterone Receptor Membrane Component 1

**DOI:** 10.3390/cancers13133265

**Published:** 2021-06-29

**Authors:** Yasuaki Kabe, Ikko Koike, Tatsuya Yamamoto, Miwa Hirai, Ayaka Kanai, Ryogo Furuhata, Hitoshi Tsugawa, Erisa Harada, Kenji Sugase, Kazue Hanadate, Nobuji Yoshikawa, Hiroaki Hayashi, Masanori Noda, Susumu Uchiyama, Hiroki Yamazaki, Hirotoshi Tanaka, Takuya Kobayashi, Hiroshi Handa, Makoto Suematsu

**Affiliations:** 1 Department of Biochemistry, Keio University School of Medicine, Tokyo 160-8582, Japan; ikko@z5.keio.jp (I.K.); mhirai@keio.jp (M.H.); kanai-a@gunma-u.ac.jp (A.K.); ryogofuruhata@keio.jp (R.F.); h.tsugawa@keio.jp (H.T.); 2 Bioorganic Research Institute, Suntory Foundation for Life Sciences (SUNBOR), 8-1-1 Seikadai, Seika, Soraku, Kyoto 619-0284, Japan; yamamoto@sunbor.or.jp (T.Y.); harada-e@sunbor.or.jp (E.H.); 3 Department of Molecular Engineering, Graduate School of Engineering, Kyoto University, Kyoto-Daigaku Katsura, Nishikyo-Ku, Kyoto 615-8510, Japan; sugase@moleng.kyoto-u.ac.jp; 4 Cokey, Co., Ltd., 2 Sanbancho, Chiyoda-ku, Tokyo 102-0075, Japan; hanadate@cokey.co.jp (K.H.); yoshikawa@cokey.co.jp (N.Y.); 5 Laboratory of Natural Products Chemistry, College of Pharmaceutical Sciences, Ritsumeikan University, 1-1-1 Nojihigashi, Kusatsu, Shiga 525-8577, Japan; hhayashi@fc.ritsumei.ac.jp; 6 U-Medico Inc., Osaka 565-0871, Japan; masanori.noda@u-medico.co.jp; 7 Department of Biotechnology, Graduate School of Engineering, Osaka University, Osaka 565-0871, Japan; suchi@bio.eng.osaka-u.ac.jp; 8 Department of Rheumatology and Allergy, IMSUT Hospital, The Institute of Medical Science, The University of Tokyo, Tokyo 108-8639, Japan; h.yamazaki66@gmail.com (H.Y.); hirotnk@ims.u-tokyo.ac.jp (H.T.); 9 Department of Medical Chemistry, Kansai Medical University, Hirakata, Osaka 573-1010, Japan; kobayatk@hirakata.kmu.ac.jp; 10 Department of Chemical Biology, Tokyo Medical University, Tokyo 160-8402, Japan; hhanda@tokyo-med.ac.jp

**Keywords:** progesterone receptor membrane component 1, EGF receptor, glycyrrhizin, glycyrrhizin derivatives, chemoresistance, low-density lipoprotein, endocytosis

## Abstract

**Simple Summary:**

Progesterone receptor membrane component 1 (PGRMC1) is highly expressed in cancer cells and enhances cancer proliferation and chemoresistance. It is therefore considered a potential target for cancer treatment. However, a chemical compound that directly regulates PGRMC1 has not been identified. Here, we showed that the natural active compound in licorice, glycyrrhizin (GL), directly binds to heme-dimerized PGRMC1 to inhibit PGRMC1-mediated EGF receptor (EGFR) activation in cancer cells. Chemical screening using GL derivatives revealed that the glucoside derivative glucoglycyrrhizin (GlucoGL) binds more potently to PGRMC1 and contributes to the suppression of PGRMC1-mediated cancer chemoresistance. This study provides the first evidence of chemical compounds that directly bind to PGRMC1 to inhibit its function, and the findings provide new insights for cancer treatments that target PGRMC1.

**Abstract:**

Progesterone receptor membrane component 1 (PGRMC1) is highly expressed in various cancer cells and contributes to tumor progression. We have previously shown that PGRMC1 forms a unique heme-stacking functional dimer to enhance EGF receptor (EGFR) activity required for cancer proliferation and chemoresistance, and the dimer dissociates by carbon monoxide to attenuate its biological actions. Here, we determined that glycyrrhizin (GL), which is conventionally used to ameliorate inflammation, specifically binds to heme-dimerized PGRMC1. Binding analyses using isothermal titration calorimetry revealed that some GL derivatives, including its glucoside-derivative (GlucoGL), bind to PGRMC1 potently, whereas its aglycone, glycyrrhetinic acid (GA), does not bind. GL and GlucoGL inhibit the interaction between PGRMC1 and EGFR, thereby suppressing EGFR-mediated signaling required for cancer progression. GL and GlucoGL significantly enhanced EGFR inhibitor erlotinib- or cisplatin (CDDP)-induced cell death in human colon cancer HCT116 cells. In addition, GL derivatives suppressed the intracellular uptake of low-density lipoprotein (LDL) by inhibiting the interaction between PGRMC1 and the LDL receptor (LDLR). Effects on other pathways cannot be excluded. Treatment with GlucoGL and CDDP significantly suppressed tumor growth following xenograft transplantation in mice. Collectively, this study indicates that GL derivatives are novel inhibitors of PGRMC1 that suppress cancer progression, and our findings provide new insights for cancer treatment.

## 1. Introduction

Progesterone receptor membrane component 1 (PGRMC1) was first identified as a membrane protein that binds to progesterone [1,2,3]. The secondary structure of PGRMC1 has a single transmembrane region at the N-terminus and a heme-binding motif in the central region, localized to the cytoplasm [4]. Several reports have shown that PGRMC1 is highly expressed in various cancer cells [4,5], and is associated with tumor progression and chemoresistance [6,7]. Several physiological functions of PGRMC1 have been suggested, including cholesterol biosynthesis [8,9], amyloid β-induced synaptotoxicity [10,11], uterine homeostasis [12], and ovarian follicle development [13]. However, these reports did not identify the molecular mechanisms through which PGRMC1 regulates cellular and organ phenotypes. We have previously reported, based on X-ray crystallographic analysis, that PGRMC1 forms a unique heme-heme stacking dimer structure through heme coordination of its Tyr113 residue [14]. The heme-dimerized PGRMC1 interacts with the EGF receptor (EGFR) and cytochromes P450 to enhance cancer proliferation and chemoresistance, respectively [14]. We have also recently shown that heme-dimerized PGRMC1 enhances lipid accumulation in adipocytes via interaction with low-density lipoprotein receptor (LDLR) or glucose transporter 4 (GLUT4) [15]. By ‘interaction’ in this manuscript, we imply the joint presence of interacting proteins in co-immunoprecipitated protein complexes, but not necessarily direct protein contacts between those proteins. Therefore, the discovery of compounds targeting heme-dimerized PGRMC1 could lead to the development of novel drugs targeting various PGRMC1 functions, including its effect on tumor progression.

Glycyrrhizin (GL) is a major active component of the herbal medicine licorice (*Glycyrrhiza glabra* or *Glycyrrhiza uralensis*) [16,17]. GL exhibits many effects, including anti-inflammatory, anti-allergic, and hepatoprotective effects [18], and is widely used in clinical practice [19]. Recently, GL has been shown to exhibit anti-cancer effects in cancers such as hepatocellular carcinoma, colon cancer, lung cancer, and breast cancer [20,21,22,23]. However, its mechanism of action remains unclear. Previously, high mobility group box 1 (HMGB1), a ubiquitous nuclear protein that is released from damaged cells and induces proinflammatory responses, was identified as a GL-binding protein that induces the anti-inflammatory action of GL [24,25]. However, the binding affinity between GL and HMGB1 is quite low, and the mechanisms underlying various physiological actions of GL have not been fully elucidated. In addition, licorice contains various GL-related derivatives [26], and the optimization of GL derivatives based on structure-activity relationships has not progressed due to insufficient receptor binding information.

We previously developed an application for high-performance affinity nanobeads, which enable direct purification of binding proteins for small-molecule compounds [27,28]. Using the beads, various receptor proteins have been identified for chemical compounds, including drugs, metabolites, and natural products [14,29,30,31,32]. In this study, we identified PGRMC1 as a pivotal target protein for GL. GL interferes with PGRMC1-mediated chemoresistance in cancer cells by inhibiting the interaction between PGRMC1 and either EGFR or LDLR. Furthermore, chemical screening using a binding assay revealed that several GL derivatives strongly inhibit PGRMC1 function. These derivatives could be useful as novel PGRMC1 inhibitors.

## 2. Materials and Methods

### 2.1. Materials

Dulbecco’s modified Eagle’s medium (DMEM), diamidino phenylindole (DAPI), CDDP, and glycyrrhizic acid dipotassium salt were purchased from Wako (Osaka, Japan). Erlotinib was purchased from Cayman Chemical (Ann Arbor MI, USA). Alexa Fluor 488 EGF complex and Alexa Fluor 488 acetylated LDL were purchased from Invitrogen (Waltham, MA, USA). Fetal bovine serum (FBS) was purchased from Biowest (Nuaille, France). FLAG and anti-FLAG antibody-conjugated agarose were purchased from Sigma-Aldrich (Burlington MO, USA). Glycyrrhetinic acid-3-*O*-mono-glucuronide and glycyrrhetinic acid monoammonium salt were purchased from Nagara Science (Gifu, Japan). Glucoglycyrrhizin [33], and rhaoglucoglycyrrhizin [26], were isolated from the roots of *Glycyrrhiza uralensis* strain 83-555, according to a previously published method by Cokey (Tokyo, Japan) [33]. Araboglycyrrhizin, apioglycyrrhizin, licorice-saponin A3, licorice-saponin G2, licorice-saponin H2, and macedonoside A were isolated from commercially available licorice extract using a similar method for GlucoGL and rhaoglucoglycyrrhizin [34]. These compounds were identified by comparing their spectral data with published data.

### 2.2. Antibodies

Antibodies were purchased from the following manufacturers: PGRMC1 (Novus Biologicals, Centennial, CO, USA: NBP1–83220), EGFR (Cell Signaling Technology, Danvers, MA, USA: #2232S), LDLR (R&D Systems, Minneapolis, MN, USA: AF2255), phospho-Y1068 EGFR (Cell Signaling Technology: #2234S), AKT (Cell Signaling Technology: #9272S), phospho-S473AKT (Cell Signaling Technology: #4060S), ERK (Cell Signaling Technology: #4695S), phospho-T185 Y187 ERK (Invitrogen: 44680 G), and CYP3A4 (Santa Cruz Biotechnology, Dallas, TX, USA: sc-390768).

### 2.3. Affinity Purification

Control and GL-or GA-fixed affinity nanobeads were prepared as previously described [14,32]. Briefly, 1 mM of either GL or GA was incubated with equal amounts of N-hydroxysuccinimide and 1-ethyl-3-(3-dimethylaminopropyl) carbodiimide (Dojindo, Kumamoto, Japan) for 2 h at room temperature, followed by overnight incubation with amino-modified affinity beads. For purification of GL or GA-binding proteins, 0.2 mg of beads were equilibrated with binding buffer (20 mM HEPES [pH 7.9], 100 mM NaCl, 1 mM MgCl_2_, 0.2 mM EDTA, 10% glycerol, 1 mM DTT, 0.2 mM PMSF, 0.1% NP40), and incubated with 1 mg/mL mouse liver extract or HCT116 cell lysate at 4 °C for 1 h. Bound proteins were eluted using SDS-loading dye, separated using SDS-PAGE, and then visualized using silver staining (Wako). Bound proteins were subjected to in-gel digestion with trypsin, and peptide fragments were analyzed using ESI-MS (Waters Corporation, Milford, MA, USA; SynaptG2).

### 2.4. Preparation of Plasmid Vectors and Recombinant Proteins

Bacterial expression vectors pGEX-PGRMC1 (human PGRMC1 intracellular domain residues 43-195) and mammalian FLAG-tagged expression vector pCMV-FLAG-PGRMC1 (full-length) were constructed as described previously [14]. Expression vectors for PGRMC1 point mutants were generated by site-directed mutagenesis with PCR. For construction of HMGB1 expression vector, human HMGB1 full-length cDNA was cloned from cDNA library of HCT116 cells using the primers (Forward: 5′-TTTGGATCCATGGGCAAAGGAGATCCTAAGAAGCC-3′, Reverse: 5′-TTTGTCGACTTATTCATCATCATC-ATCTTCTTC-3′), digested with Bam HI and Sal I, and then ligated into pGEX6P1.

Expression vectors (pGEX-PGRMC1 (residues 43-195) or pGEX-HMGB1) were transformed into BL21 (DE3) competent *E. coli* cells, and the cells incubated in LB medium with ampicillin at 37 °C until the OD_600_ reached 0.8. Protein expression was induced by adding 1 mM isopropyl-β-thiogalactopyranoside (IPTG) and incubating at 37 °C for 4 h. After harvesting cells, the cell pellets were then resuspended in buffer containing 20 mM Tris-HCl (pH 7.5), 100 mM NaCl, and 0.1% Tween 20, sonicated twice at 4 °C for 5 min, and centrifuged at 20,000× *g* for 30 min. The supernatant was incubated with glutathione Sepharose 4B (GE Healthcare, Chicago, IL, USA) for 1 h at 4 °C. The resin was then washed five times with the same buffer, and the GST tag cleaved by adding Precision Protease (GE Healthcare) and incubating at 4 °C for 16 h. Apo-PGRMC1 or HMGB1 proteins was purified using size-exclusion chromatography in a Superdex 200 column (GE Healthcare). Heme-bound PGRMC1 was prepared by treating with 100 μM hemin and then purified using size-exclusion chromatography. 

Isotope-labelled PGRMC1 proteins for NMR analyses were prepared in the same manner, except that the cells were grown in minimal M9 media in H_2_O or 99.9% ^2^H_2_O, including ampicillin, metals, vitamins, ^15^N-ammonium chloride, and ^13^C or ^12^C glucose as sources of nitrogen and carbon, respectively. Protein expression was induced by adding 1 mM IPTG and incubating at 20 °C for 40 h. Protein purification was performed as described above. The GST tag fused to the N-terminus of PGRCM1 was cleaved using Factor Xa (GE Healthcare).

### 2.5. ITC Analyses

ITC experiments were performed at 298 K with ITC-Buffer (50 mM phosphate [pH 7.0]) using a MicroCal iTC200 (Malvern Panalytical, Malvern, UK). GL derivatives were dissolved in 3 mM ITC-Buffer and titrated into 100 μM apo-PGRMC1, heme-bound-PGRMC1, or HMGB1 protein. Titration was performed by injecting 2 µL of the syringe solution at intervals of 120 s. Binding isotherms were analyzed using SEDPHAT [35]. The binding of AG205 to apo- or heme-PGRMC1 (100 μM) was analyzed using a concentration of 3 mM containing 1% DMSO. The binding affinities of GL derivatives for HMGB1 (80 µM) were analyzed using a concentration of 3 mM.

### 2.6. NMR Analyses

For NMR measurements, 500 μM of GL-bound or heme-dimerized PGRMC1 were prepared in 50 mM phosphate buffer (pH 7.0) containing 5% D_2_O. NMR spectra were measured at 25 °C using a Bruker Avance DRX 600 spectrometer equipped with a triple resonance (^1^H/^13^C/^15^N) cryogenic probe. Transverse relaxation-optimized spectroscopy (TROSY) types of 2D ^1^H-^15^N heteronuclear single-quantum coherence (HSQC) spectra were measured. NMR data were processed using the program NMRPipe [36], and signal assignments were performed using the programs Kujira [37], and NMRView [38]. A docking model structure of the GL-bound PGRMC1 was calculated using the program HADDOCK 2.1 [39] using the crystal structure of PGRMC1 (PDB: 4X8Y) and the chemical shift data, and residues showing Δ*δ* larger than 0.05 ppm were defined as the GL-binding site.

### 2.7. Hydrogen Deuterium Exchange Mass Spectrometry (HDX-MS)

HDX-MS experiments were conducted using a Waters HDX with a LEAP system (Waters Corporation). The PGRMC1+GL complex solution was prepared by mixing equal volumes of 160 μM PGRMC1 solution and 8 mM GL solution. The 80 μM protein solutions (PGRMC1 and PGRMC1+GL complex) were diluted 20-fold in 50 mM phosphate buffer (pH 7.0) prepared with D_2_O containing 150 mM NaCl and incubated at 20 °C for various hydrogen/deuterium exchange time points (0.5, 1, 10, 60, or 240 min). The exchange reaction was quenched by lowering the pH to 2.4, which was achieved by mixing with an equal volume of 4 M guanidinium chloride 0.5 M tris(2-carboxyethyl) phosphine hydrochloride (TCEP) (pH 2.2). Quenched samples (100 pM) were immediately injected, desalted, and separated online using a Waters UPLC system based on the nanoACQUITY platform. Online digestion was performed over 6 min in water containing 0.05% formic acid at 4 °C at a flow rate of 100 μL/min. The digested peptides were trapped on an ACQUITY UPLC BEH C18 1.7 μm peptide trap (Waters Corporation) maintained at 0 °C and desalted with water and 0.1% formic acid. Flow was diverted using a switching valve, and the trapped peptide fragments were eluted at 40 μL/min onto a column of 1 × 100 mm (C18 (1.7 μm), ACQUITY UPLC BEH, Waters Corporation) held at 0 °C, with a 9 min linear acetonitrile gradient (8–40%) containing 0.1% formic acid. The eluate was directed into a Synapt HD mass spectrometer (Waters Corporation) with electrospray ionization and lock mass correction (using Glu-fibrinogen peptide B). Mass spectra were transformed using MassLynx (Waters Corporation) and acquired over an m/z range of 100–2000. Pepsin fragments were identified using a combination of exact mass and MS/MS aided by ProteinLynx Global SERVER (PLGS, Waters Corporation). Peptide deuterium levels were determined using DynamX 3.0 (Waters Corporation). Peptides that were significantly different between PGRMC1 and PGRMC1+GL complexes were evaluated by creating volcano plots with 99% confidence intervals for the degree of deuterium exchange [40]. Reliable identification of significant differences in differential hydrogen exchange–mass spectrometry measurements was analyzed using a hybrid significance testing approach.

### 2.8. Cell Lines and Culture

The HCT116 cells, a human colon cancer cell line, and HuH7 cells, a human hepatoma cell line, were maintained in Dulbecco’s modified Eagle’s medium (DMEM) containing 10% fetal bovine serum (FBS) and 1% penicillin-streptomycin at 37 °C in a 5% CO_2_ humidified incubator. The PGRMC1 knockdown (KD) cell line was established in a previous study using lentivirus vectors [14].

For analysis of EGFR signaling, cells were incubated overnight with GL or GlucoGL, and then EGF (100 ng/mL) was added for 5 min. Cells were lysed with RIPA buffer, and the lysates were subjected to SDS-PAGE and visualized through western blotting using antibodies against PGRMC1, EGFR, phospho-Y1068 EGFR, AKT, phospho-S473AKT, ERK, and phospho-T185 Y187 ERK. 

For the analysis of EGF and LDL uptake, HCT116 cells were incubated with serum-free DMEM supplemented with Alexa Fluor 488 EGF complex or Alexa Fluor 488 acetylated LDL to a final concentration of 1 µg/L at 37 °C for 5 min. For immunofluorescence microscopy analyses, HCT116 cells treated with Alexa Fluor 488-labeled EGF or LDL were fixed with 4% paraformaldehyde for 20 min and incubated in 10 mg/L DAPI for 15 min. The cells were observed using a BZ-x800 fluorescence microscope (Keyence, IL, USA). For flow cytometry analysis, HCT116 cells treated with Alexa Fluor 488-labeled EGF or LDL were detached using trypsin/EDTA and suspended in PBS. The mean fluorescent intensity per 10,000 cells was analyzed using a flow cytometer (Gallios, Beckman Coulter Life Science, Pasadena, CA, USA). The data were analyzed using Kaluza 2.1 analysis software (Beckman Coulter Life Science).

To analyze the chemosensitivity of HCT116 or HuH7 cells, the cells were incubated with erlotinib (20 µM) or CDDP (10 µM) and either GL or GL derivatives for 24 h on a 96-well plate. Cell viability was determined using an MTT assay kit (Millipore, Burlington, MA, USA) according to the manufacturer’s instructions.

### 2.9. Co-Immunoprecipitation Assays

An expression vector of pCMV-3xFLAG-PGRMC1 or an empty vector was transfected into HCT116 cells or HuH7 cells using the transfection reagent Lipofectamine 2000 (Invitrogen). The cells were incubated overnight with either GL or GlucoGL. Cells were then lysed with NP40 lysis buffer (20 mM Tris-HCl [pH 7.5], 150 mM NaCl, and 1% NP40). The lysates were incubated with 10 μL of equilibrated anti-FLAG (M2) agarose for 60 min at room temperature. Bound proteins were washed three times and subjected to SDS-PAGE and visualized through western blotting using antibodies against FLAG, EGFR, and LDLR. For analysis of the endogenous PGRMC1-binding with FLAG-PGRMC1, HCT116 cells (1 × 10^7^ cells) were treated with vehicle or 250 μM SA and/or 10 μM hemin for 48 h, and the cell lysates were co-immunoprecipitated with anti-FLAG (M2) agarose. Bound proteins (endogenous PGRMC1 and FLAG-tagged PGRMC1) were visualized through western blotting using antibody against PGRMC1.

For in vitro binding assays between PGRMC1 and CYP3A4, 1 μg of purified CYP3A4 proteins (Sigma: C4982) were incubated with 10 μg of FLAG-PGRMC1 (a.a. 44-195) treated with or without GL or GlucoGL in 500 μL of binding buffer containing 20 mM HEPES-NaOH (pH 7.9), 100 mM NaCl, 0.2 mM EDTA, 10% glycerol, and 0.1% NP40 for 60 min at room temperature. Then, 10 μL of equilibrated anti-FLAG (M2) agarose was added to the mixture, which was then incubated for 60 min at room temperature. Bound proteins were washed three times with 200 μL of binding buffer and eluted with 10 μL of 2 μg/mL FLAG elution peptide (Sigma: F4799). The eluates were subjected to SDS–PAGE and visualized by Western blotting using antibodies against CYP3A4 (Santa Cruz: sc-53850) and FLAG.

### 2.10. EGFR Kinase Assay

The assay was performed using an EGFR kinase assay kit (BPS Bioscience, San Diego, CA, USA). EGFR kinase activity was analyzed by measuring the amount of ATP remaining following the kinase reaction based on luminescent intensity using Kinase-Glo MAX (Promega, Madison, WI, USA). A mixture of kinase buffer, ATP, and PTK substrate was incubated with EGFR (1 ng/µL) with or without GL or GlucoGL at 30 °C for 40 min. Kinase-Glo max was added to each well and incubated at room temperature for 15 min under light protection. Luminescent intensity was measured using a microplate reader.

### 2.11. Xenograft Implantation of HCT116 Cells

All protocols for animal experiments in this study were approved by the Experimental Animal Committee of Keio University School of Medicine (approval number: 08024-(13), approval date: 30 September 2020). HCT116 cells (2 × 10^6^ cells/mouse) with half of Matrigel (Corning, NY, USA) were transplanted subcutaneously into the flank of 7-week-old nude mice. Four days after the transplantation, tumor size was measured in three dimensions with calipers and grouped by tumor volume. CDDP (2 mg/kg), GL (400 mg/kg), or GlucoGL (100 mg/kg) were administered to the mice intraperitoneally, and the tumor volume was measured twice a week for 22 days.

### 2.12. Statistical Analysis

All statistical analyses were performed using BellCurve for Excel (SSRI, Tokyo, Japan). Experimental results are presented as the mean ± standard error (S.E.). Student’s *t*-test was used for two-group comparisons, and Dunnett’s test was used for multiple comparisons. Differences were considered statistically significant at *p* values < 0.05. Experiments using the cell lines were conducted in triplicate.

## 3. Results

### 3.1. Identification of PGRMC1 as a Novel GL-Binding Protein

**Figure 1:**
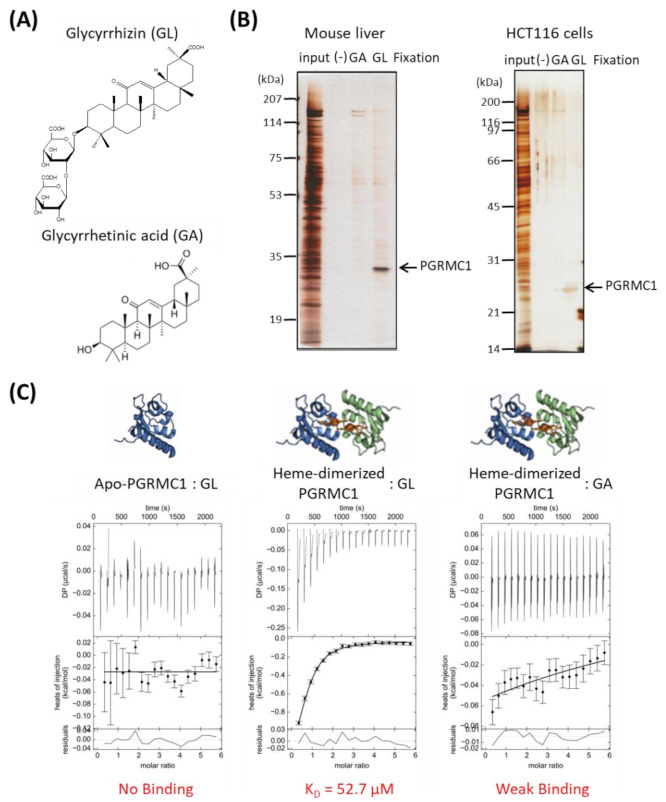
Identification of Progesterone receptor membrane component 1 (PGRMC1) as a glycyrrhizin (GL)-binding protein. (**A**) Chemical structures of GL and glycyrrhetinic acid (GA). (**B**) Affinity screening of the GL-binding protein. The affinity nanobeads fixed with control, (-), GA or GL were incubated with lysates derived from mouse liver (left panel) or HCT116 cells (right panel), and bound proteins were analyzed using SDS Polyacrylamide gel electrophoresis (SDS-PAGE) and visualized using silver staining. The protein of interest (indicated by the arrow) was identified as PGRMC1 through peptide sequencing using electrospray ionization mass spectrometry (ESI-MS). (**C**) Analysis of the binding affinity between PGRMC1 and GL or GA using isothermal titration calorimetry (ITC). The binding affinity of GL was analyzed using apo- (left panel) or heme-dimerized PGRMC1 (middle panel) with ITC analyses. The right panel shows binding between GA and heme-dimerized PGRMC1.

We performed affinity screening to identify the GL-binding protein. Using magnetic affinity nanobeads conjugated with GL or its aglycone, glycyrrhetinic acid (GA), (Figure 1A), binding proteins were purified from lysates prepared from mouse liver or human colon cancer HCT116 cells. Among several observed proteins, one with a molecular weight of approximately 25 kDa in the mouse liver and HCT116 cell lysates bound specifically to the GL-beads (Figure 1B). This protein was identified as PGRMC1 using electrospray ionization mass spectrometry (ESI-MS) analysis.

We analyzed the binding affinity between GL and PGRMC1 using isothermal titration calorimetry (ITC). We previously showed that PGRMC1 is converted from a monomer to a dimer by binding to heme [14]. ITC analyses revealed that GL bound to the heme dimeric form of PGRMC1, but not to the apo-monomer form, with a binding affinity (K_D_) of 52.70 μM (Figure 1C). We did not detect significant binding between GA and heme-dimerized PGRMC1 using ITC analysis. These results indicate that triterpenoid saponin GL specifically binds to the heme-dimerized PGRMC1.

### 3.2. Binding Structure of GL to Heme-Dimerized PGRMC1

We previously analyzed the soluble structure of apo- and heme-dimerized PGRMC1 using nuclear magnetic resonance (NMR) based on the X-ray crystallographic structure of PGRMC1 (PDB: 4X8Y) [14]. To reveal the site of binding between GL and heme-dimerized PGRMC1, we measured two-dimensional transverse relaxation-optimized spectroscopy (TROSY) heteronuclear single-quantum coherence (HSQC) spectra of the GL-bound or non-bound PGRMC1 using NMR. The chemical shift differences between the GL-bound and non-bound forms of the heme-dimerized PGRMC1 were calculated (Figure S1) using previously assigned chemical shifts of the heme-dimerized PGRMC1 [14]. Note that the ^1^H and ^15^N chemical shifts in the GL-bound form were shifted by *J*_HN_/2 because a regular HSQC spectrum was measured for the heme-dimerized PGRMC1 without GL, whereas a TROSY HSQC spectrum was measured for the GL-bound form, and TROSY HSQC signals were generally measured at the ^1^H chemical shift of *J*_HN_/2 smaller values and ^15^N chemical shift of *J*_HN_/2 larger values compared with the regular HSQC. The weighted chemical shift difference (∆δ) between the apo- and GL-bound forms of PGRMC1 was calculated as:

$$∆\delta =\sqrt{{\left(∆\delta_{H}\right)}^{2}+{\left(∆\delta_{N}/5\right)}^{2}\;}$$

where Δ*δ*_H_ and Δ*δ*_N_ are the chemical shift differences in the ^1^H and ^15^N nuclei, respectively.

**Figure 2:**
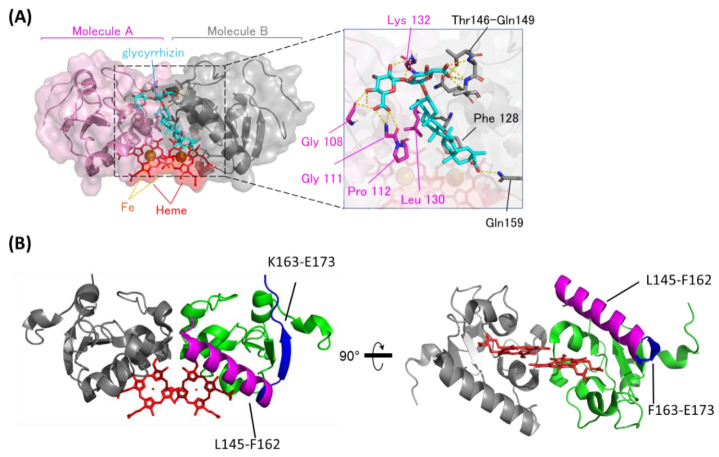
The binding structure between GL and heme-dimerized PGRMC1. (**A**) A model structure of GL-bound PGRMC1 generated in the HADDOCK program using the crystal structure (PDB: 4X8Y) and Nuclear Magnetic Resonance (NMR) chemical shift perturbation data (Figure S1). PGRMC1 molecules (pink and gray) are dimerized by binding to heme molecules (red). A GL molecule (light blue) binds to the dimer interface of PGRMC1 molecules. The triterpene of GL is positioned in proximity to the heme molecules. (**B**) Hydrogen/deuterium exchange mass spectrometry (HDX-MS) results of PGRMC1 and GL-bound PGRMC1 complex. The data show more protected regions (L145-F162; magenta) and more exposed regions (F163-E173; blue) in GL-bound PGRMC1 complex compared with PGRMC1.

Based on the chemical shift changes caused by binding to GL, the GL/heme-dimerized PGRMC1 binding model structure was constructed using the biomolecular interaction software HADDOCK2.1 [39]. As shown in the GL-binding model structure (Figure 2A), GL is positioned at the dimer interface of two PGRMC1 molecules generated by heme-mediated dimerization. The GL triterpene site binds to hydrophobic regions via PGRMC1 residues Pro112, Phe128, and Leu130, and the GL glucuronic acids moiety bind to PGRMC1 via hydrogen bonds on residues Gly108, Gly111, Lys132, and Thr146–Gln149. We confirmed this model structure using PGRMC1 point mutations in residues near the putative GL-binding site: G108W, G111W, F128W, K132V, T146W, A147W, and Q149F. GL-binding affinity for PGRMC1 with these point mutations was decreased compared with wildtype PGRMC1 (Figure S2 and Table S1), and the binding affinity for G108W, A147W, and Q149F PGRMC1 mutants was significantly decreased. These residues suggest that the hydrophilic interaction between GL glucuronic acid and PGRMC1 is important for its affinity.

We also confirmed the GL-binding region in heme-dimerized PGRMC1 by hydrogen deuterium exchange mass spectrometry (HDX-MS) analysis (Figure 2B and Figure S3). HDX-MS detected two regions of PGRMC1 with different degrees of deuterium exchange in the presence of GL, and one region: L145–F162, showed a decrease in deuterium exchange with the addition of GL, implying the protection of these region. The other region: F163–E173, showed an increase in the deuterium exchange rate. These results suggest that PGRMC1 L145–F162 residues located in the α-helix which forms a groove through heme dimerization is the GL-binding site in PGRMC1.

### 3.3. Binding Analyses Using GL Derivatives

**Figure 3:**
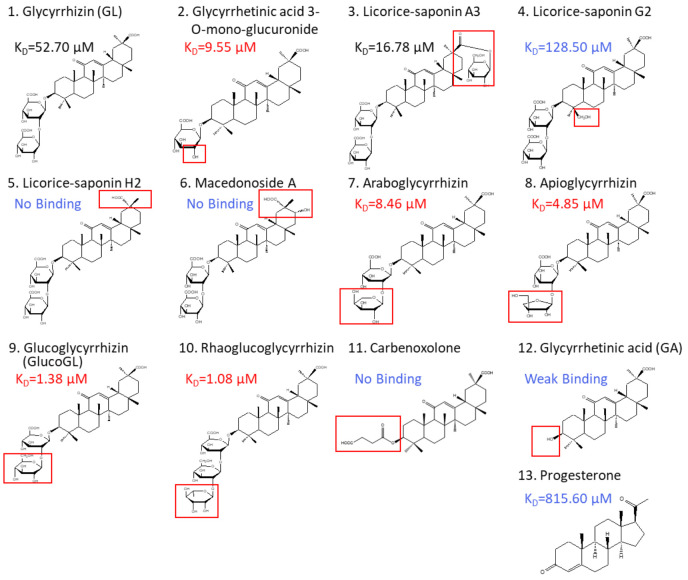
Chemical structures of GL derivatives examined in this study. The red squares show the structural regions that differ from GL. Binding affinities (K_D_) for PGRMC1 were analyzed using ITC.

*Glycyrrhiza uralensis* contains several glycyrrhizin derivatives. We prepared and analyzed GL-related derivatives, as shown in Figure 3. Binding analyses using ITC revealed that the binding affinity for PGRMC1 was reduced or abolished when using GL derivatives, such as licorice-saponin G2, licorice-saponin H2, or macedonoside A that were converted at the triterpene regions (Table 1, Figure S4). Furthermore, GL aglycone derivatives, such as carbenoxolone or GA, also had lower binding affinity compared with GL. In contrast, PGRMC1 binding affinity was enhanced when using a derivative from which the second glucuronic acid in GL was removed or converted to a neutral sugar, for example glycyrrhetinic acid 3-*O*-mono-glucuronide, araboglycyrrhizin, apioglycyrrhizin, glucoglycyrrhizin (GlucoGL), or rhaoglucoglycyrrhizin. Notably, the GL-glucoside derivatives, GlucoGL, and the rhamnoside derivative, rhaoglucoglycyrrhizin, exhibited high binding affinities (K_D_ = 1.38 μM and 1.08 μM, respectively). It is possible that these derivatives may be more effective GL-related compounds. In addition, PGRMC1 was originally identified as a progesterone-binding protein [3]. ITC analysis showed weak binding between progesterone and PGRMC1 (K_D_ = 815.60 μM), suggesting that progesterone has no physiologically effect on PGRMC1. AG205 is considered a PGRMC1 inhibitor [41]. We analyzed the binding between AG205 and PGRMC1, but no binding activity to apo- or heme-dimerized PGRMC1 was observed by ITC analysis (Figure S5A). We also analyzed GL-binding affinity for HMGB1, which has previously been reported to bind to GL [24]. The binding affinity of GL was lower for HMGB1 than for PGRMC1 (K_D_ = 222.94 μM), and weak binding was observed when GlucoGL was used (Figure S5B). These results suggest that GL derivatives could potently bind to heme-dimerized PGRMC1 to exhibit their functions.

### 3.4. GL Derivatives Interfere with EGFR Signaling by Inhibiting the Interaction between PGRMC1 and EGFR

We previously showed that heme-dimerized PGRMC1 enhances EGFR signaling by binding to EGFR [14]. Previous studies indicate that PGRMC1 forms a dimer in several cells [42,43,44,45]. We confirmed the intracellular dimerization of PGRMC1 by binding to heme in HCT116 cells. Ectopically expressed FLAG-tagged PGRMC1 in HCT116 cells was immunoprecipitated with anti-FLAG antibody, and bound endogenous PGRMC1 was detected by western blotting (Figure S6). Endogenous PGRMC1 (25 kDa) was co-immunoprecipitated with FLAG-PGRMC1 (30 kDa), and this binding was attenuated by treatment with SA, an inhibitor of heme biosynthesis [46]. This binding inhibition by SA was canceled by treatment of hemin, suggesting that PGRMC1 forms stable complex by binding with heme in cells. In addition, treatment with GL did not affect the PGRMC1-PGRMC1 binding. These results suggested that PGRMC1 forms the heme-mediated dimer in HCT116 cells.

**Figure 4:**
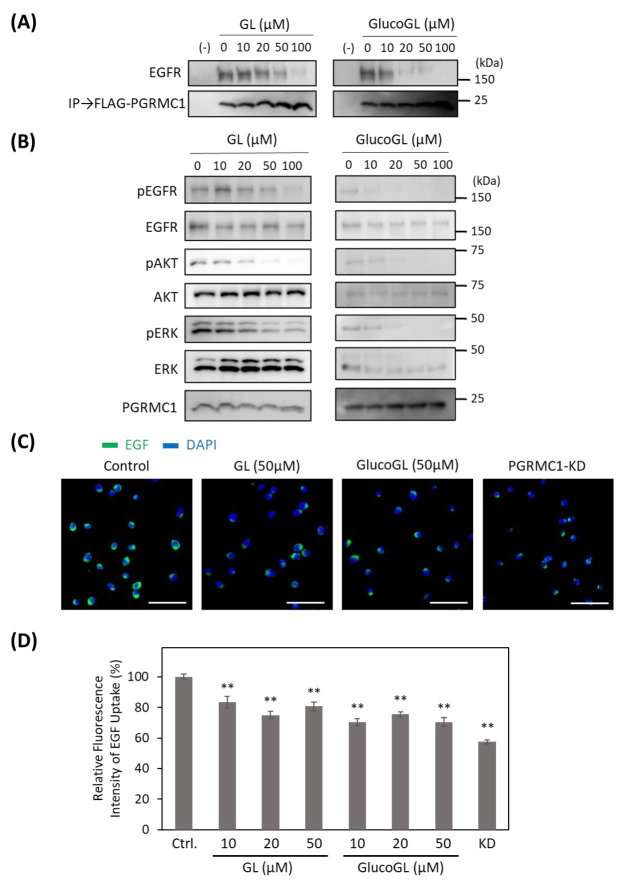
GL derivatives inhibit the interaction between PGRMC1 and EGF receptor (EGFR), and suppress EGFR signaling. (**A**) Co-immunoprecipitation assay of the binding between PGRMC1 and EGFR. FLAG-PGRMC1 was overexpressed in HCT116 cells treated for 12 h with either GL or GlucoGL. The cell lysates were immunoprecipitated using anti-FLAG antibody-fixed beads. Co-immunoprecipitated proteins (FLAG-PGRMC1 and EGFR) were detected through western blotting using anti-FLAG or anti-EGFR antibodies. (**B**) HCT116 cells treated with either GL or GlucoGL were incubated with EGF for 5 min, and components of the EGFR signaling pathway were detected using western blotting. (**C**) HCT116 cells treated with or without GL, GlucoGL, or PGRMC1-knockdown (KD) cells were incubated with Alexa Flour 488 EGF complex. Fluorescence microscopic images of the incorporated EGF (green) and diamidino phenylindole (DAPI) (blue) are shown (Scale bar; 100 μm). (**D**) Flowcytometric analysis of EGF uptake in HCT116 cells. HCT116 cells treated with or without GL, GlucoGL, or PGRMC1-KD cells were incubated with Alexa Flour 488 EGF complex for 5 min. The bars show the mean fluorescence intensities (per 10,000 cells) (*n* = 6). Data represent the mean ± SE. Statistical analysis was performed using Dunnett’s test. ** *p* < 0.01.

To determine the effect of GL derivatives on PGRMC1 function, we performed a co-immunoprecipitation assay for the interaction between PGRMC1 and EGFR in HCT116 cells (Figure 4A).

Endogenous EGFR was co-immunoprecipitated using FLAG-tagged PGRMC1, and its binding was inhibited by treatment with GL. GlucoGL treatment inhibited the interaction more potently. We also showed that GL and GlucoGL inhibited the interaction between PGRMC1 and EGFR in human hepatoma HuH7 cells (Figure S7A). Similarly, EGF-induced EGFR signaling, such as phosphorylation of EGFR, AKT, and ERK, was significantly suppressed by treatment with either GL or GlucoGL (Figure 4B). We also performed time-course analyses of EGF signaling with or without treatment of GlucoGL (Figure S8), and GlucoGL showed the remarkable inhibitory effect in the early phase of EGF stimulation (5 or 10 min). Furthermore, treatment of GL or GlucoGL also suppressed the EGF signaling in HuH7 cells (Figure S7B). It has been reported that some GL derivatives bind to EGFR to inhibit its phosphorylation activity [47]. We examined the effect of GL and GlucoGL on EGFR kinase activity in vitro (Figure S9). While the EGFR inhibitor erlotinib potently inhibited kinase activity, no inhibitory effect was observed when GL or GlucoGL were used. EGFR carries out its function via endocytosis by binding to EGF [48]. PGRMC1 regulates the endocytic pathway, including EGFR and LDLR [15,49]. We analyzed the effect of GL derivatives on the uptake of the Alexa Flour 488 EGF complex into HCT116 cells using immunofluorescence microscopy and flow cytometry (Figure 4C,D). The uptake of labelled EGF in cells treated with GL, GlucoGL, or PGRMC1-knockdown (KD) was significantly suppressed compared with control cells. These results suggest that GL derivatives inhibit the interaction between PGRMC1 and EGFR, and subsequently suppress EGFR signaling and EGFR-mediated endocytosis.

### 3.5. GL Derivatives Interfere with LDL Uptake by Inhibiting the Interaction between PGRMC1 and LDLR

**Figure 5:**
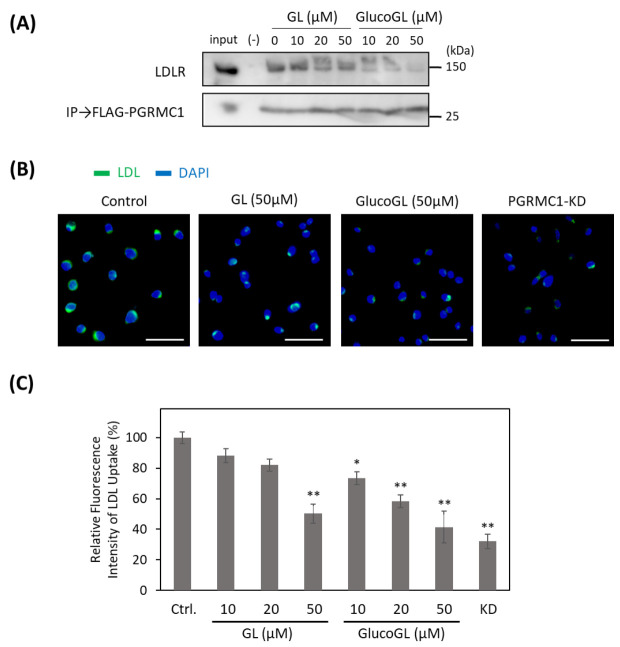
GL derivatives inhibit the interaction between PGRMC1 and low-density lipoprotein receptor (LDLR) and suppress LDL uptake. (**A**) Co-immunoprecipitation assay of the binding between PGRMC1 and LDLR. FLAG-PGRMC1 was overexpressed in HCT116 cells treated for 12 h with either GL or GlucoGL. The cell lysates were immunoprecipitated using anti-FLAG antibody-fixed beads. Co-immunoprecipitated proteins (FLAG-PGRMC1 and LDLR) were detected through western blotting using anti-FLAG or anti-LDLR antibodies. (**B**) HCT116 cells treated with GL, GlucoGL, or PGRMC1-KD cells were incubated with Alexa Fluor488-labelled LDL. Fluorescence microscopic images of the incorporated LDL (green) and DAPI (blue) are shown (Scale bar; 100 μm). (**C**) Flowcytometric analysis of LDL uptake in HCT116 cells. HCT116 cells treated with GL, GlucoGL, or PGRMC1-KD cells were incubated with Alexa Fluor488-labeled LDL for 5 min. The bars show the mean fluorescence intensities (per 10,000 cells) (*n* = 6). Data represent the mean ± SE. Statistical analysis was performed using Dunnett’s test. * *p* < 0.05 or ** *p* < 0.01.

Recent reports have shown that PGRMC1 contributes to cellular LDL uptake by regulating LDLR [15,50]. We used the co-immunoprecipitation assay to examine the interaction between PGRMC1 and LDLR in HCT116 cells (Figure 5A). Endogenous LDLR was co-immunoprecipitated using FLAG-tagged PGRMC1, and its binding was inhibited by treatment with GL. GlucoGL treatment inhibited this interaction more potently. Furthermore, we analyzed the effect of GL derivatives on Alexa Fluor 488-labelled LDL uptake in HCT116 cells using immunofluorescence microscopy and flow cytometry (Figure 5B,C). Labelled-LDL uptake was significantly suppressed by treatment with GL, GlucoGL or PGRMC1-KD. These results suggest GL derivatives inhibit the interaction between PGRMC1 and LDLR, and subsequently suppress LDLR-mediated endocytosis. In addition, we previously showed heme-dimerized PGRMC1 interacts with cytochrome P450 3A4 (CYP3A4) [14]. Co-immunoprecipitation assays revealed that GL and GlucoGL interfered with the interaction between PGRMC1 and CYP3A4 (Figure S10). These results suggest GL derivatives inhibit the interaction between PGRMC1 and its interactors, such as EGFR, LDLR, and CYP3A4, by binding to the heme-dimeric structure of PGRMC1.

### 3.6. GL Derivatives Enhance Chemosensitivity in Cancer Cells

**Figure 6:**
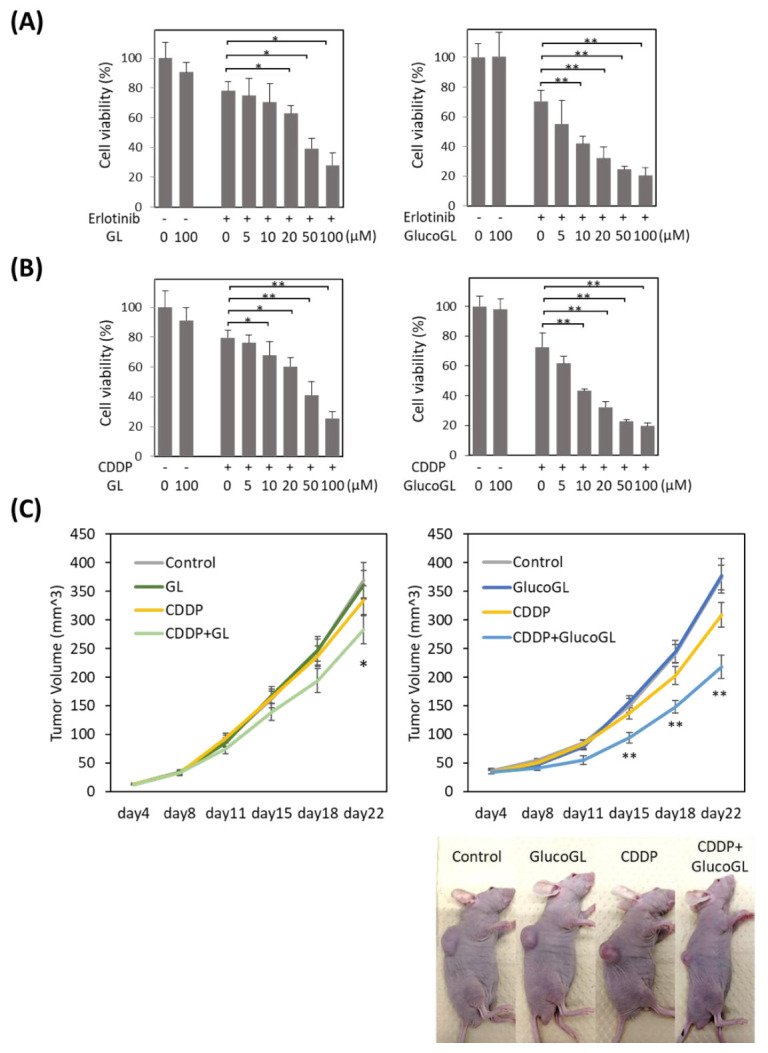
GL derivatives enhance chemosensitivity in HCT116 cells. Erlotinib (**A**) or cisplatin (CDDP) (**B**) was incubated for 12 h with HCT116 (control) cells treated with GL or GlucoGL, and cell viability was examined using MTT assay. The data represent the mean ± SE of three separate experiments. * *p* < 0.05 or ** *p* < 0.01 using Student’s *T*-test. (**C**) HCT116 (control) cells were injected subcutaneously into the flanks of each nude mouse to initiate tumor growth for 22 days. The mice were then intraperitoneally treated with either control vehicle, CDDP (2 mg/kg), GL (400 mg/kg), or CDDP (2 mg/kg) and GL (400 mg/kg) (left panel), or CDDP (2 mg/kg), GlucoGL (100 mg/kg) or CDDP (2 mg/kg) and GlucoGL (100 mg/kg) (right panel) twice a week for a total of 22 days. Tumor volume was measured twice a week in three dimensions throughout the study. Data represent the mean ± SE of three separate experiments. * *p* < 0.05 and ** *p* < 0.01 using Dunnett’s test.

We previously showed that suppression of PGRMC1 through knockdown significantly enhanced the cytotoxicity of cancer cells by anti-cancer agents, including the EGFR inhibitor erlotinib [14]. We analyzed the effect of GL derivatives on cancer cell proliferation. The anti-cancer agents, erlotinib and cisplatin (CDDP), were administered to HCT116 cells treated with or without GL derivatives, and cell viability was evaluated using MTT assay (Figure 6A,B, Figures S11 and S12). Although no effect was observed following treatment with GL derivatives in the absence of anti-cancer agents, GL treated with erlotinib or CDDP significantly suppressed cell viability, with half maximal inhibitory concentrations (IC_50_) of 51.99 µM and 53.07 µM, respectively (Table 1). Treatment with GlucoGL showed a more potent antiproliferative effect (IC_50_ = 15.67 µM and 14.92 µM, respectively). Furthermore, GL derivatives that strongly bound to PGRMC1, such as araboglycyrrhizin, apioglycyrrhizin, and rhaoglucoglycyrrhizin, showed potent anti-cancer effects. In contrast, when GL derivatives with low binding affinity for PGRMC1, such as licorice-saponin G2, licorice-saponin H2, macedonoside A, or carbenoxolone, were used, weaker anti-proliferative effects compared with the effect of GL were observed. We also showed that GL and GlucoGL treated with erlotinib or CDDP significantly suppressed cell viability in HuH7 cells (Figure S13). These results showed that the anti-cancer activity of GL derivatives was correlated with the binding affinities of GL derivatives for PGRMC1.

We further analyzed the effect of GL derivatives on a subcutaneous transplant model of cancer cells in nude mice. HCT116 cells were subcutaneously transplanted into the flank of nude mice. CDDP, GL, GlucoGL, or CDDP combined with either GL (400 mg/kg) or GlucoGL (100 mg/kg) was intraperitoneally injected twice a week, and the tumor volume measured (Figure 6C).

When CDDP or GL were administered individually, there were no differences in tumor volume compared with the control. When CDDP and GL were used in combination, the tumor volume was significantly smaller compared with that of the control 22 days after transplantation (Figure 6C, left panel). Furthermore, administration of CDDP and GlucoGL in combination significantly suppressed the tumor volume from 15 days after transplantation (Figure 6C, right panel). GlucoGL strongly suppressed tumor volume, even at 1/4 the concentration of GL. These results indicate that GL enhances the effect of anti-cancer drugs in vivo, and GlucoGL exhibits a more potent effect.

## 4. Discussion

In this study, we determined that GL derivatives specifically bind to PGRMC1 and act as its inhibitors that suppress PGRMC1-mediated function. PGRMC1 was originally identified as a membrane protein that binds to the endogenous hormone progesterone [1,2,3]. GL is a saponin consisting of a steroid-related triterpene structure that conjugates two glucuronic acids. Analysis of the GL-bound PGRMC1 structure revealed that GL selectively binds to the groove on PGRMC1 that is generated through its heme dimerization. The GL triterpene region exhibited hydrophobic interactions with this groove, and the GL sugar chain retained its binding activity through hydrophilic interactions with PGRMC1 (Figure 2). Supporting this, the GL aglycone derivatives GA or carbenoxolone disrupted any binding activity with PGRMC1 (Table 1), and PGRMC1 residue mutations such as G108W or Q149F, which are required for hydrogen bonding to the GL sugar chain, significantly reduced the binding affinity (Table S1). On the other hand, progesterone is reported to recognize several membrane proteins, including the adipoQ receptor (PAQR) family of progestin receptors (mPRs) [51]. Furthermore, a competitive binding assay using labeled-progesterone suggests that a GFP-fused PGRMC1 protein partially purified from granulosa cells binds to progesterone with high affinity (K_D_ = 35 nM) [52]. However, these studies provide no evidence that progesterone binds directly to PGRMC1. In our ITC analysis, we observed low-affinity binding (K_D_ = 815 μM) between progesterone and heme-dimerized PGRMC1. These results suggested that progesterone has a very low affinity for the PGRMC1 cytoplasmic region, and it may bind with a hydrophobic environment containing PGRMC1 and cell membrane, or with a membrane protein complex with PGRMC1 such as sigma-2 receptor TMEM97, or an interacting factor(s) of PGRMC1. In contrast, we found that some derivatives, such as araboglycyrrhizin, apioglycyrrhizin, GlucoGL, or rhaoglucoglycyrrhizin, in which the glucuronic acid moiety of GL is replaced with a neutral sugar, showed higher affinity for PGMRC1 than GL (Table 1). Although the exact reason for this remains unclear, analyses of the binding affinities of these derivatives for PGRMC1 using ITC showed that their Gibbs free energy (ΔG) was significantly higher than that of GL (Table 1), suggesting that replacement of the carboxylic acid group of GL glucuronic acid with a neutral group enhanced the binding affinity. This information will be useful for the development of further derivatives targeting PGRMC1. In addition, we did not observe AG205 binding to apo- or heme-dimerized PGRMC1 (Figure S5A). AG205 was originally identified as a compound that bound to Arabidopsis PGRMC1 homolog *At2g24940* [53]; however, in mammalian PGRMC1, there is no direct evidence that AG205 has an affinity for binding to PGRMC1. A recent study showed that AG205 affected the formation of large vesicular structures independent of PGRMC1 using PGRMC1 knockout cells [54], indicating that AG205 acts through a target different from PGRMC1. Further analysis is necessary to examine how AG205 exerts its pharmacological actions.

We have shown that heme-dimerized PGRMC1 interacts with EGFR, LDLR, and cytochromes P450 to regulate their functions [14,15]. In this study, we showed that GL derivatives interfere with these interactions by binding to heme-dimerized PGRMC1. Although the exact mechanism of protein interaction induced by PGRMC1 heme dimerization is unclear, the PGRMC1 grooves generated through heme dimerization may contribute to these interactions, and GL derivatives likely inhibit these interactions by filling the grooves. We showed that GL derivatives suppressed PGRMC1-mediated EGFR signaling and EGF or LDL uptake (Figure 4 and Figure 5). Besides heme binding with PGRMC1, recent report shows that PGRMC1 Y113 residue, which is essential for heme binding, is phosphorylated in breast cancer MCF7 cells [55]. The functional regulation of PGRMC1 by this phosphorylation has remained unclear, but the PGRMC1 function may be regulated by varying heme binding state through changes in the phosphorylation and de-phosphorylation status of the Y113 residue. PGRMC1 is thought to play an important role in regulating intracellular protein translocation, as PGRMC1 contains several YXXϕ motifs that are implicated in vesicle transport and endocytosis [4,5,56]. Therefore, our results suggest that GL derivatives would interfere with the endocytosis of EGF or LDL by inhibiting the interaction between PGRMC1 and EGFR/LDLR. We have previously shown that heme-dimerized PGRMC1 increases cancer resistance against anti-cancer drugs in HCT116 cells [14]. Similar to those results, in this study, GL enhanced the sensitivity of the drugs erlotinib and CDDP, and the neutral sugar-containing derivatives such as GlucoGL showed even stronger anti-cancer activity compared with that of GL (Figure 6, Figures S8 and S9). The GlucoGL-binding affinity (K_D_) with PGRMC1 was 1.38 µM, while the cell growth inhibitory concentration of GlucoGL with the addition of erlotinib or CDDP (IC_50_ = 15.67µM or 14.92 µM, respectively) was weaker than that shown by its K_D_ value (Table 1). This may be due to the low intracellular incorporation of these GL derivatives. Although the exact mechanism by which GL derivatives enhance the anticancer effect of CDDP is unclear, it has been known that activation of EGFR contributes to enhance anti-apoptotic effect by chemotherapeutic agents [57,58,59], therefore, the suppression of EGF signaling by GL derivatives mediated by PGRMC1 might enhance the anti-cancer effect of CDDP. We also showed that GL derivatives inhibit PGRMC1-mediated LDL uptake (Figure 5). LDL contains a large portion of cholesterol and plays an important role in tumor growth, including membrane lipid synthesis, and tumor growth is suppressed by statins [60]. GL has several effects, including degradation of lipid rafts and micelle and fibril networks formation [61]. Inhibition of PGRMC1 by GL derivatives may be involved in reducing the amount of LDL uptake into cells to suppress tumor growth.

In addition to its anti-cancer effects, GL reportedly has multiple functions, including anti-inflammatory, anti-allergic, and hepatoprotective functions [18]. HMGB1 has been identified as a binding target for GL [24]. HMGB1 is released from damaged cells and acts as a circulating damage-associated molecular pattern (DAMP) molecule that induces inflammatory, autoimmune, and cardiovascular diseases or neurological disorders [62]. GL is thought to block HMGB1-mediated alarmin function [63]. In this study, we showed that GL binding to heme-dimerized PGRMC1 was 4.23 times stronger than its binding to HMGB1. In agreement with this, GL, at approximately 50 μM, suppressed PGRMC1-mediated EGFR activation and LDL uptake, enhancing anti-cancer activity. Furthermore, although no binding activity was observed between HMGB1 and GlucoGL, which binds strongly to PGRMC1, GlucoGL exhibited a more potent anti-cancer effect in vitro and in vivo (Figure 6). These results indicate that the GL derivative used in this study showed anti-cancer effects by inhibiting PGRMC1 function, independent of HMGB1. On the other hand, although inhibition of PGRMC1-mediated EGFR signaling by GL derivatives contributes significantly to their suppression of cancer growth and chemotherapeutic resistance, PGRMC1 is known to regulate various functional pathways, including the insulin response and heme biosynthesis pathways [64,65]. Thus, it is possible that GL derivatives exhibit anticancer effects through pleiotropic actions mediated by these pathways. Our analyses also revealed that GL derivatives inhibit interactions between PGRMC1 and LDLR or the drug-metabolizing enzyme, CYP3A4, suggesting that GL derivatives may synergize with other anticancer drugs via PGRMC1. Because GL exhibits pleiotropic effects, including anti-inflammatory and hepatoprotective effects, it will be necessary to comprehensively evaluate the mechanisms by which GL derivatives exhibit anticancer effects using gene expression analyses in future cancer cell studies. In addition, several reports have suggested that PGRMC2, a PGRMC1 paralog, contributes to heme biosynthesis and brown adipocyte differentiation co-operatively with PGRMC1 [65,66]. PGRMC2 has also been hypothesized to form a heme-mediated heterocomplex with PGRMC1 [66]. In addition to PGRMC2, membrane-associated progesterone receptor family proteins (MAPR), such as neuferricin and neudesin, which share recognizable homology to PGRMC1, are known to be functionally regulated by heme binding [67]. Although further analysis is necessary, analyses of the binding of heme-bound MAPR proteins with GL derivatives (or its absence) may help elucidate the pleiotropic actions of GL.

There are some limitations to the current study. (1) Further analyses are needed to evaluate whether the PGRMC1-mediated anti-cancer effect of GL derivatives drives EGFR and LDLR activation by PGRMC1 and other effects, including the regulation of cytochromes P450. (2) Further elucidation of the structure-activity relationship of organically synthesized GL derivatives is needed. (3) Further evaluation is also needed to determine whether the diverse functions of GL, such as anti-inflammatory effects, are mediated by PGRMC1, HMGB1, or other targets. (4) To evaluate the comprehensive mechanism of anticancer effects of GL derivatives mediated by PGRMC1 is needed by global gene expression and protein abundance analysis in cancer cells. (5) Further elucidation is needed to determine the types of cancer cells to which the PGRMC1-mediated anti-cancer activity of GL derivatives can be applied. (6) In this study, we showed the interaction with ectopic expressed FLAG-PGRMC1, but further analysis is necessary for detecting the interaction with endogenous PGRMC1 by using crosslinkers. (7) Further evaluation of the enhancing effect of various anticancer agents including erlotinib by GL derivatives in mouse models is needed.

**Figure 7:**
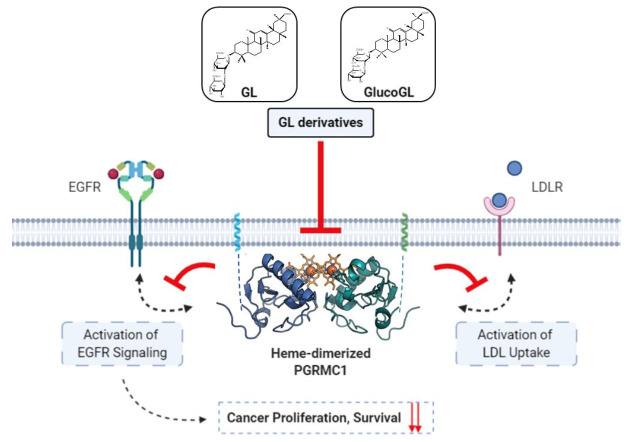
Schematic model showing the inhibition of PGRMC1-mediated function by GL derivatives in cancer cells. Heme-dimerized PGRMC1 contributes to cancer proliferation and chemoresistance via activation of EGFR signaling and LDL uptake through direct interaction with EGFR or LDLR. GL derivatives specifically bind to heme-dimerized PGRMC1 and inhibit the interaction of PGRMC1 with EGFR or LDLR, thereby suppressing PGRMC1-mediated cancer proliferation and chemoresistance.

In this study, we identified PGRMC1 as a novel binding protein for GL and showed that GL exhibits its mediated anti-cancer effect by inhibiting PGRMC1 function, including EGFR activation and LDL uptake. Based on the binding activity of heme-dimerized PGRMC1, chemical screening with a natural product library revealed that several derivatives, including GlucoGL, exhibited more potent anti-cancer effects than GL (Figure 7). While PGRMC1 has attracted a lot of attention as a therapeutic target for regulating a variety of disease conditions such as dementia and adipogenesis [11,15], pharmacological interventions for controlling them remain to be developed. Because GL has already been used clinically to treat inflammation and hepatitis, its safety has been established in humans. In our analyses, treatment with the GL derivative alone had no effect on cell viability, but it significantly enhanced the anticancer activity of chemotherapeutic agents such as erlotinib and CDDP. Therefore, adjunctive combination therapy using GL derivatives has the potential to be an effective treatment for refractory cancers, without serious adverse effects. This study is the first to analyze direct evidence of the binding affinity and structure-activity relationship between chemical compounds and PGRMC1, and this information will provide new insights for the evaluation of GL function and discovery of drugs targeting PGRMC1 function.

## 5. Conclusions

This study shows that GL derivatives are novel compounds for directly binding with PGRMC1 to inhibit its anti-cancer function. The enhancing effect of chemotherapeutic agents by treatment of GL derivatives may lead to the development of new anticancer therapy strategies.

## Figures and Tables

**Table 1:** Binding affinity of GL derivatives with PGRMC1.

Compound	K_D_ (μM)	ΔG (kcal/mol)	ΔH (kcal/mol)	TΔS (K·kcal/mol)	IC 50 (μM)	
					Erlotinib	CDDP
Glycyrrhizin (GL)	52.70	−5.84	−1.23	4.61	51.99	53.07
Glycyrrhetinic acid-3-O-mono-glucuronide	9.55	−6.85	−0.62	6.23	50.50	41.23
Licorice-saponin A3	16.78	−6.52	−1.66	4.86	40.03	39.44
Licorice-saponin G2	128.50	−5.31	−1.23	4.08	ND	ND
Licorice-saponin H2	No Binding				ND	ND
Macedonoside A	No Binding				ND	ND
Araboglycyrrhizin	8.46	−6.93	−1.03	5.90	35.30	26.58
Apioglycyrrhizin	4.85	−7.26	−1.08	6.18	62.48	35.15
Glucoglycyrrhizin (GlucoGL)	1.38	−8.00	−0.56	7.44	15.67	14.92
Rhaoglucoglycyrrhizin	1.08	−8.15	−2.07	6.08	19.05	17.25
Carbenoxolone	No Binding				ND	ND
Glycyrrhetinic acid (GA)	Weak Binding				ND	ND
Progesterone	815.60	−4.22	−23.26	−19.04	ND	ND

## Data Availability

The data presented in this study are available in the article or the Supplementary Materials.

## Supplementary Materials

The following are available online at https://www.mdpi.com/article/10.3390/cancers13133265/s1, Figure S1: Chemical shift perturbation in PGRMC1 caused by GL binding, Figure S2: Raw data of ITC analysis of the binding between GL and PGRMC1 mutants, Figure S3: HDX analysis of GL-PGRMC1 binding, Figure S4: Raw data of ITC analyses of the binding between GL derivatives and heme-dimerized PGRMC1, Figure S5: ITC analysis of the binding of high-mobility group box 1 (HMGB1) to GL, and PGRMC1 to AG205, Figure S6: The effect of the heme-dimerization of PGRMC1 by SA, Figure S7: The effect of GL derivatives to the EGF signaling in HuH7 cells, Figure S8: Time-course analyses of the effect of GlucoGL to EGF signaling in HCT116 cells, Figure S9: The in vitro effect of EGFR kinase on GL derivatives, Figure S10: GL derivatives inhibit the interaction between PGRMC1 and cytochrome P450 3A4 (CYP3A4) in vitro, Figures S11 and S12: The effect of GL derivatives on erlotinib/CDDP sensitivity in HCT116 cells, Figure S13: The effect of GL derivatives on erlotinib/CDDP sensitivity in HCT116 cells Figure S14: Full images of western blots, Table S1: Binding affinities of PGRMC1 mutants for GL based on ITC analysis.
