# Supplementary material for: Glycyrrhizin Derivatives Suppress Cancer Chemoresistance by Inhibiting Progesterone Receptor Membrane Component 1
Source: Cancers (Basel). 2021 Jun 29;13(13):3265. doi: 10.3390/cancers13133265 (PMC8269059; doi:10.3390/cancers13133265)
Supplement: Supplementary file 1 [file cancers-13-03265-s001.zip › cancers-1158611-supplementary.pdf]

cancers

Supplementary Materials

Glycyrrhizin derivatives suppress cancer chemoresistance by inhibiting Progesterone Receptor Membrane Component 1

Yasuaki Kabe, Ikko Koike, Tatsuya Yamamoto, Miwa Hirai, Ayaka Kanai, Ryogo Furuhata, Hitoshi Tsugawa, Erisa Harada, Kenji Sugase, Kazue Hanadate, Nobuji Yoshikawa, Hiroaki Hayashi, Masanori Noda, Susumu Uchiyama, Hiroki Yamazaki, Hirotoshi Tanaka, Takuya Kobayashi, Hiroshi Handa, Makoto Suematsu

Table S1. Binding affinities of PGRMC1 mutants for GL based on ITC analysis

Mutant	K_D (μM)	ΔG (kcal/mol)	ΔH (kcal/mol)	TΔS (K·kcal/mol)
**WT**	52.70	−5.84	−1.23	4.61
**G108W**	185.25	−5.09	−3.51	1.58
**G111W**	108.05	−6.25	−1.86	4.39
**F128W**	137.25	−5.27	−4.90	0.38
**K132V**	84.62	−5.56	−1.01	4.55
**T146W**	97.90	−4.81	−2.17	2.64
**A147W**	157.95	−5.19	−3.09	2.10
**Q149F**	402.82	−3.75	−14.37	−10.62

Copyright: © 2021 by the authors. Licensee MDPI, Basel, Switzerland. This article is an open access article distributed under the terms and conditions of the Creative Commons Attribution (CC BY) license (http://creativecommons.org/licenses/by/4.0/).

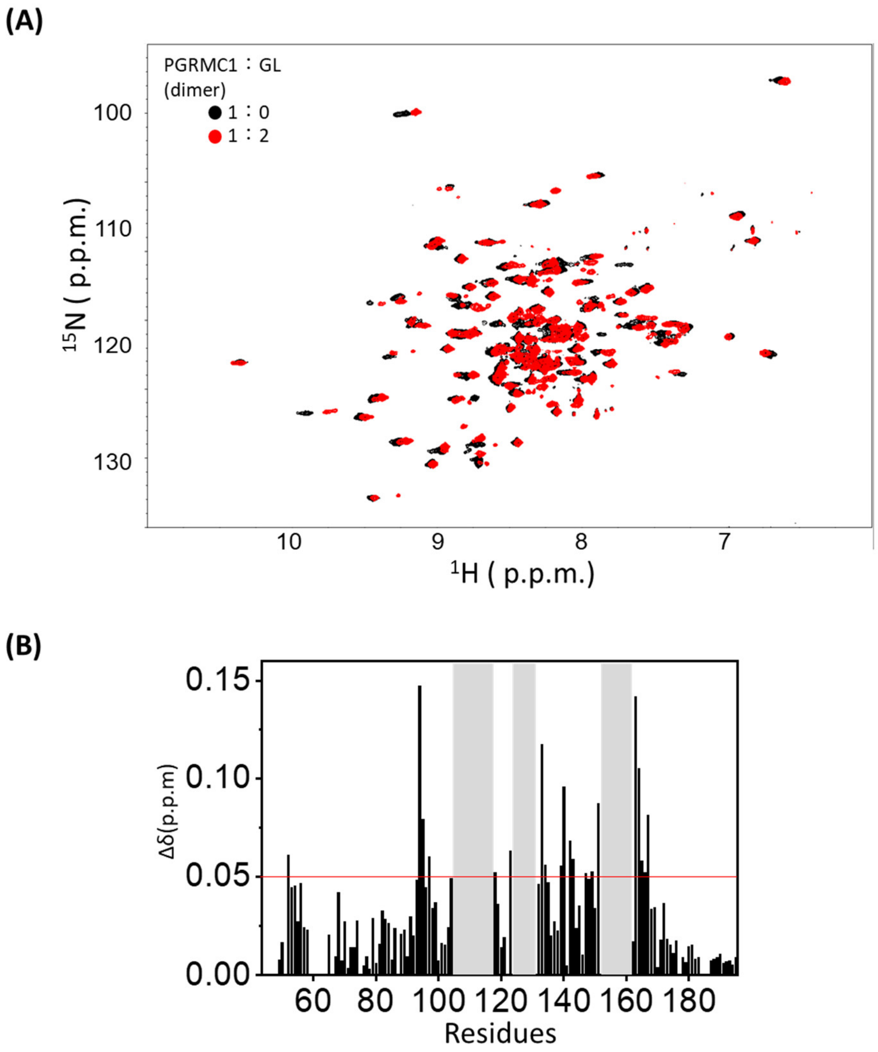

Figure S1. Chemical shift perturbation in PGRMC1 caused by GL binding. (A) [15N, 1H]- transverse relaxation-optimized spectroscopy (TROSY) spectra of the apo and GL-bound forms of PGRMC1. (B) Chemical shift changes in heme-dimerized PGRMC1 caused by binding of GL at a molar ratio of heme-dimerized PGRMC1:GL of 1:2. NMR signals of the residues in the gray regions were unobservable because of the paramagnetic relaxation effect from heme iron. The residues showing chemical shift differences larger than 0.05 ppm were defined as the binding sites in the calculation of the GL-bound PGRMC1 structure using HADDOCK software.

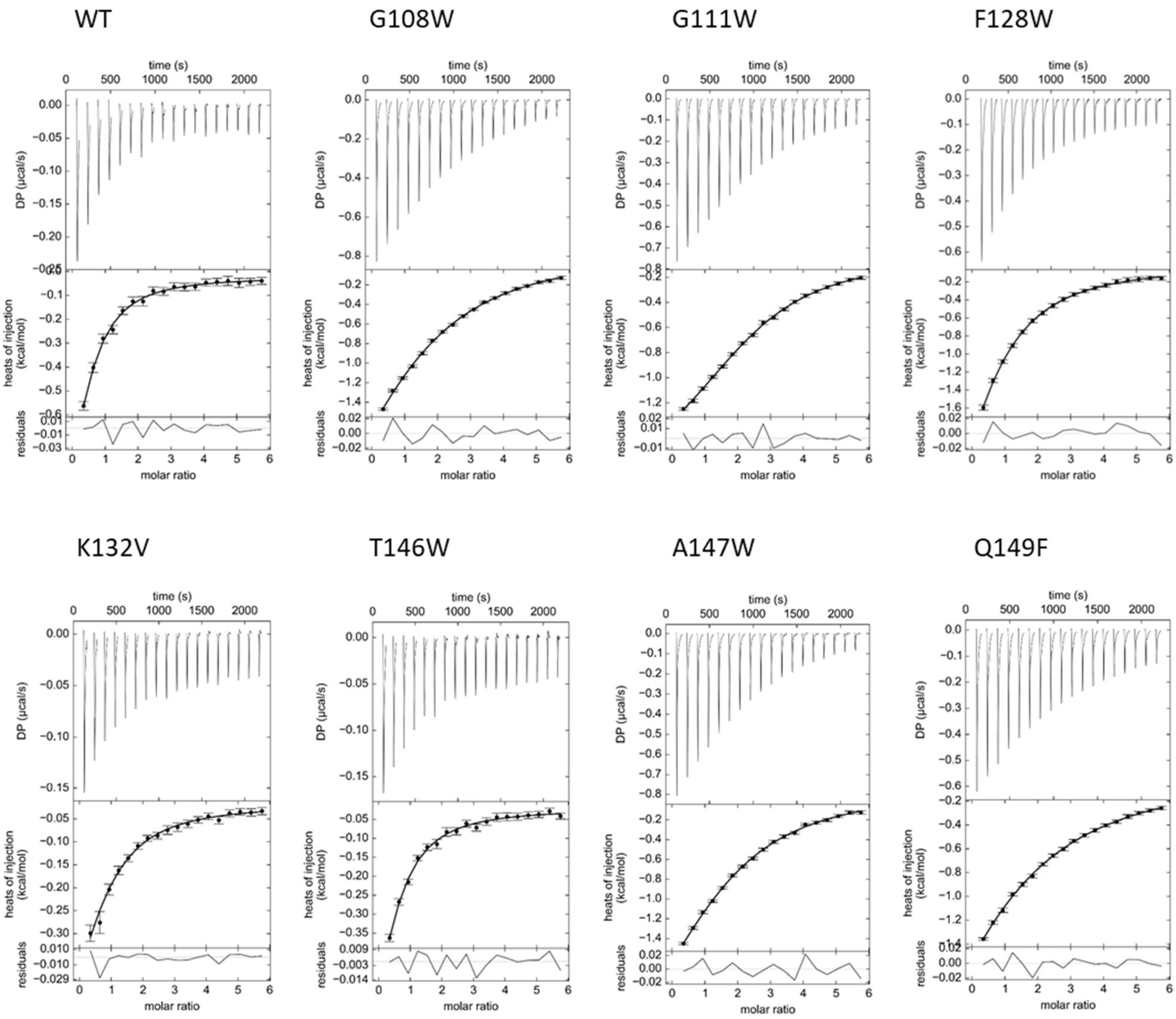

Figure S2. Raw data of ITC analyses of the binding between GL and PGRMC1 mutants.

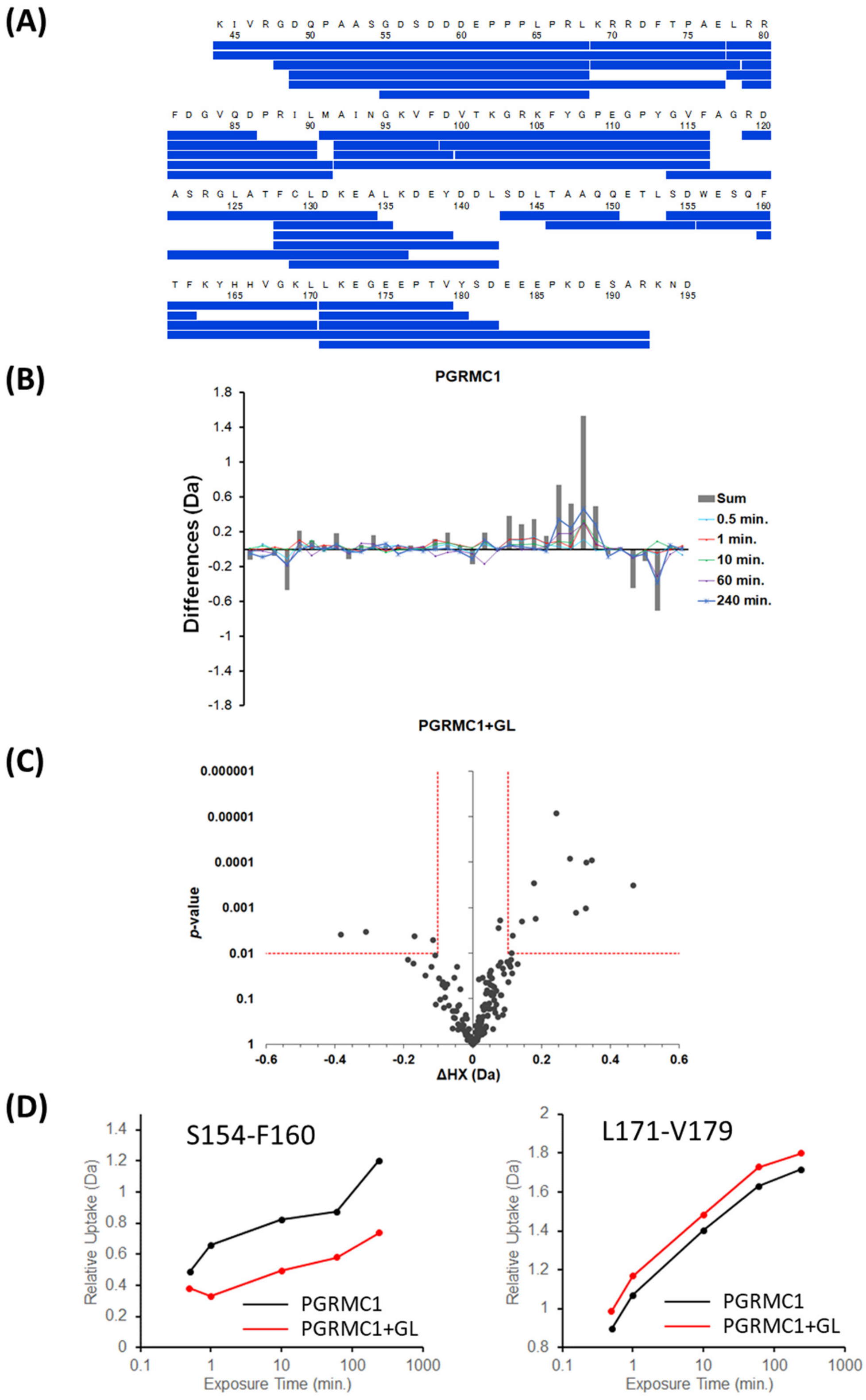

Figure S3. HDX analysis of GL-PGRMC1 binding. (**A**) Coverage map of peptides identified in PGRMC1 proteins through HDX analysis. (**B**) Differential plots of the degrees of deuterium peptide uptake, showing time course, along with their summation results (gray bar). (**C**) Volcano plots of observed ΔHX values. Red lines represent the horizontal p-value and represent the vertical ΔHX values for the significant criteria. PGRMC1 p-values calculated using Welch's *t*-test. (**D**) Deuterium uptake curves of the peptides showing significant differences between PGRMC1 (black) and PGRMC1+GL complex (red) proteins are presented.

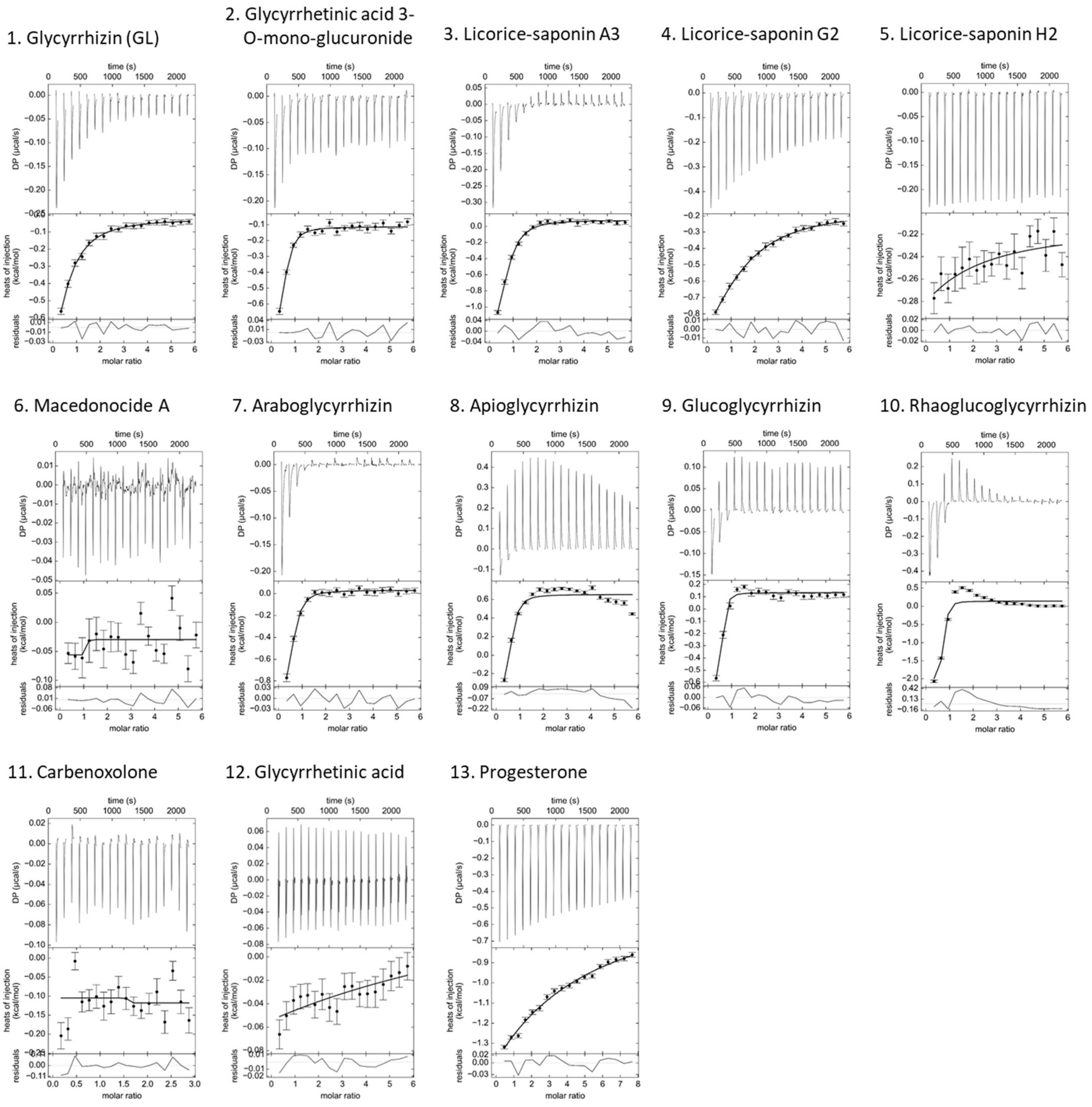

Figure S4. Raw data of ITC analyses of the binding between GL derivatives and heme-dimerized PGRMC1.

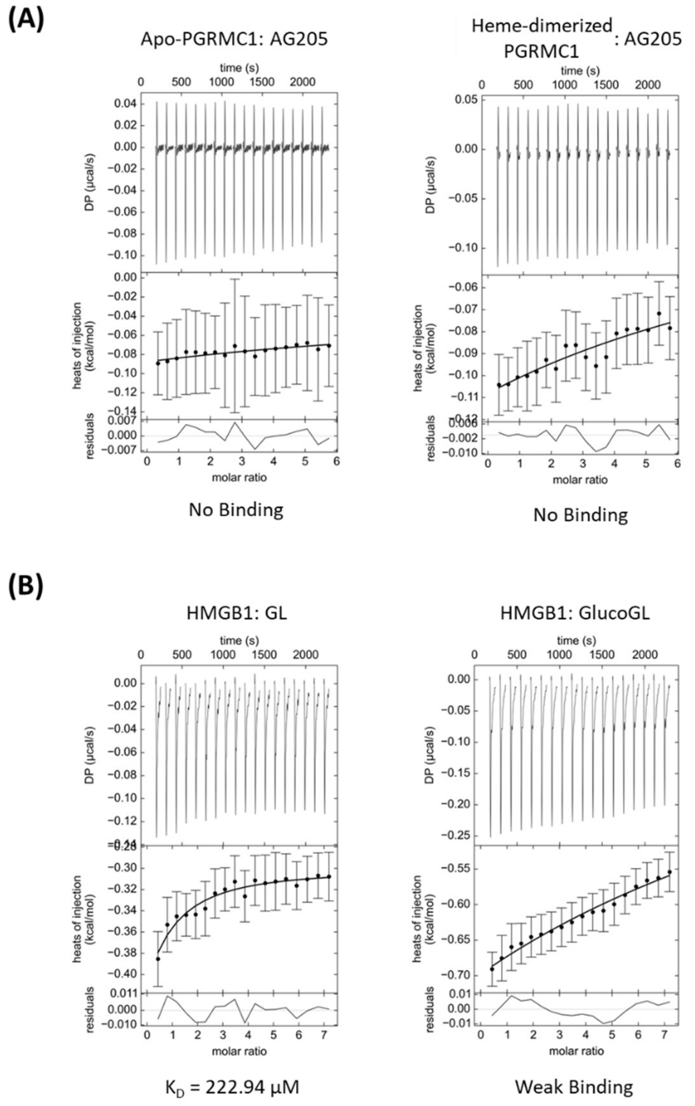

Figure S5. ITC analysis of the binding of high-mobility group box 1 (HMGB1) to GL, and PGRMC1 to AG205. (**A**) ITC analyses of the binding of apo-PGRMC1 (left) or heme-dimerized PGRMC1 (right) to AG205. (**B**) ITC analyses of the binding of high-mobility group box 1 (HMGB1) to GL (left) or GlucoGL (right).

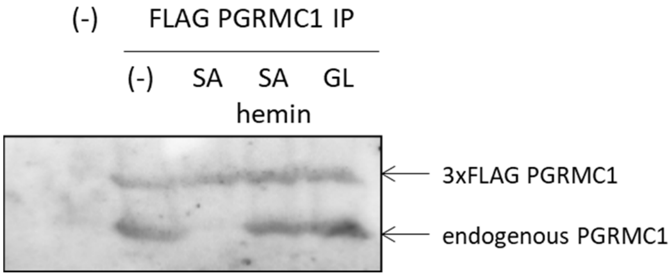

Figure S6. Co-immunoprecipitation assay of the binding between endogenous PGRMC1 and FLAG-PGRMC1. FLAG-PGRMC1 was overexpressed in HCT116 cells treated with or without 250 μM succinylacetone (SA) and/or 10 μM hemin for 48 h, or 50 μM GL for 12 hr. The cell lysates were immunoprecipitated using anti-FLAG antibody-fixed beads. Co-immunoprecipitated proteins (FLAG-PGRMC1 and endogenous) were detected through western blotting using anti-PGRMC1 antibodies.

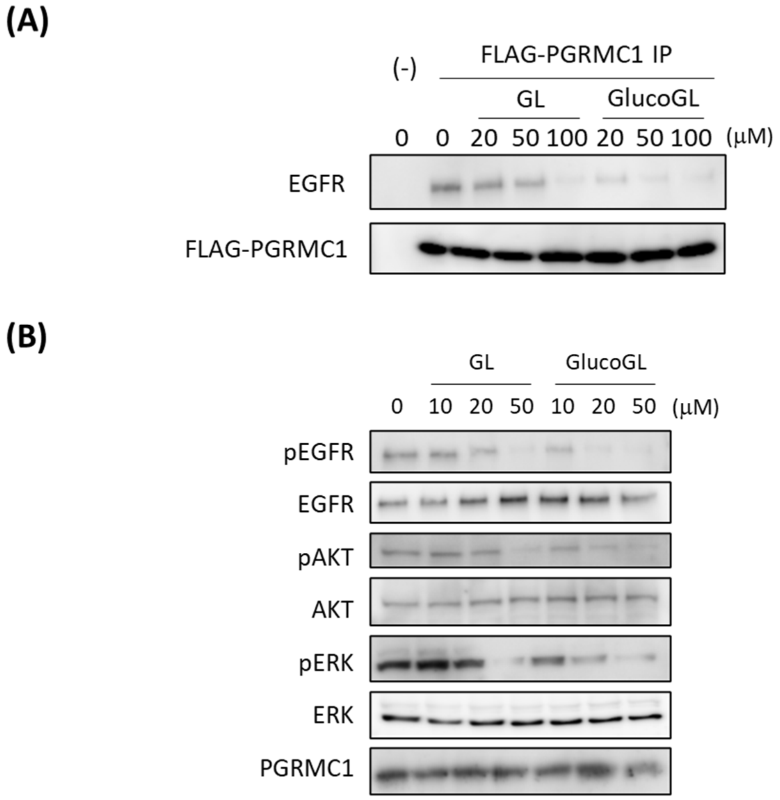

Figure S7. GL derivatives suppress the EGF signaling in HuH7 cells. (**A**) Co-immunoprecipitation assay of the binding between PGRMC1 and EGFR. FLAG-PGRMC1 was overexpressed in HuH7 cells treated for 12 h with either GL or GlucoGL. The cell lysates were immunoprecipitated using anti-FLAG antibody-fixed beads. Co-immunoprecipitated proteins (FLAG-PGRMC1 and EGFR) were detected through western blotting using anti-FLAG or anti-EGFR antibodies. (**B**) HuH7 cells treated with either GL or GlucoGL were incubated with EGF for 5 min, and components of the EGFR signaling pathway were detected using western blotting.

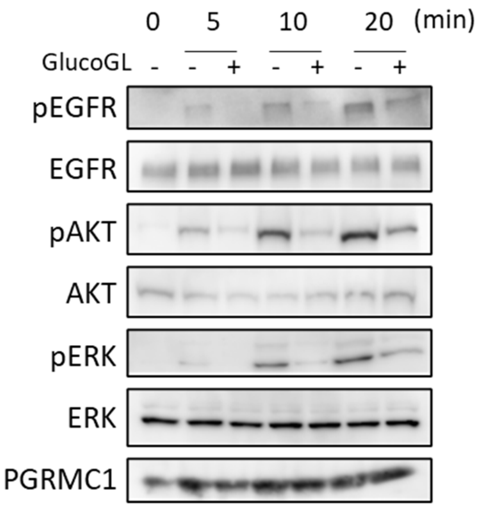

Figure S8. Time-course analyses of the effect of GlucoGL to EGF signaling in HCT116 cells. HCT116 cells treated with 20 μM GlucoGL were incubated with EGF for 5, 10 or 10 min, and components of the EGFR signaling pathway were detected using western blotting.

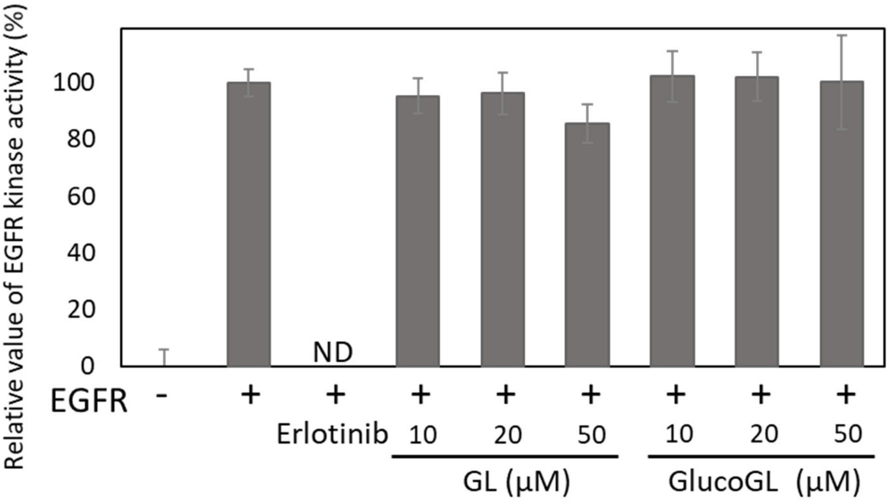

Figure S9. The in vitro effect of EGFR kinase on GL derivatives. EGFR kinase activity was evaluated by measuring the amount of remaining ATP post kinase reaction, with or without the addition of erlotinib (100nM), GL, or GlucoGL. The data represent the mean ± SE of relative value of EGFR kinase activity (*n* = 6). Statistical analysis was performed using Dunnett's test.

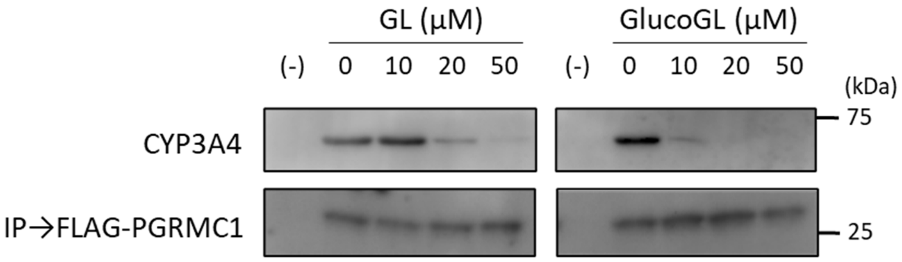

Figure S10. GL derivatives inhibit the interaction between PGRMC1 and cytochrome P450 3A4 (CYP3A4) in vitro. Purified FLAG-PGRMC1 was incubated with CYP3A4 protein in the presence of either GL or GlucoGL. FLAG-PGRMC1 was immunoprecipitated using anti-FLAG antibody-fixed beads. Co-immunoprecipitated proteins (FLAG-PGRMC1 and CYP3A4) were detected through western blotting using anti-FLAG or anti-CYP3A4 antibodies.

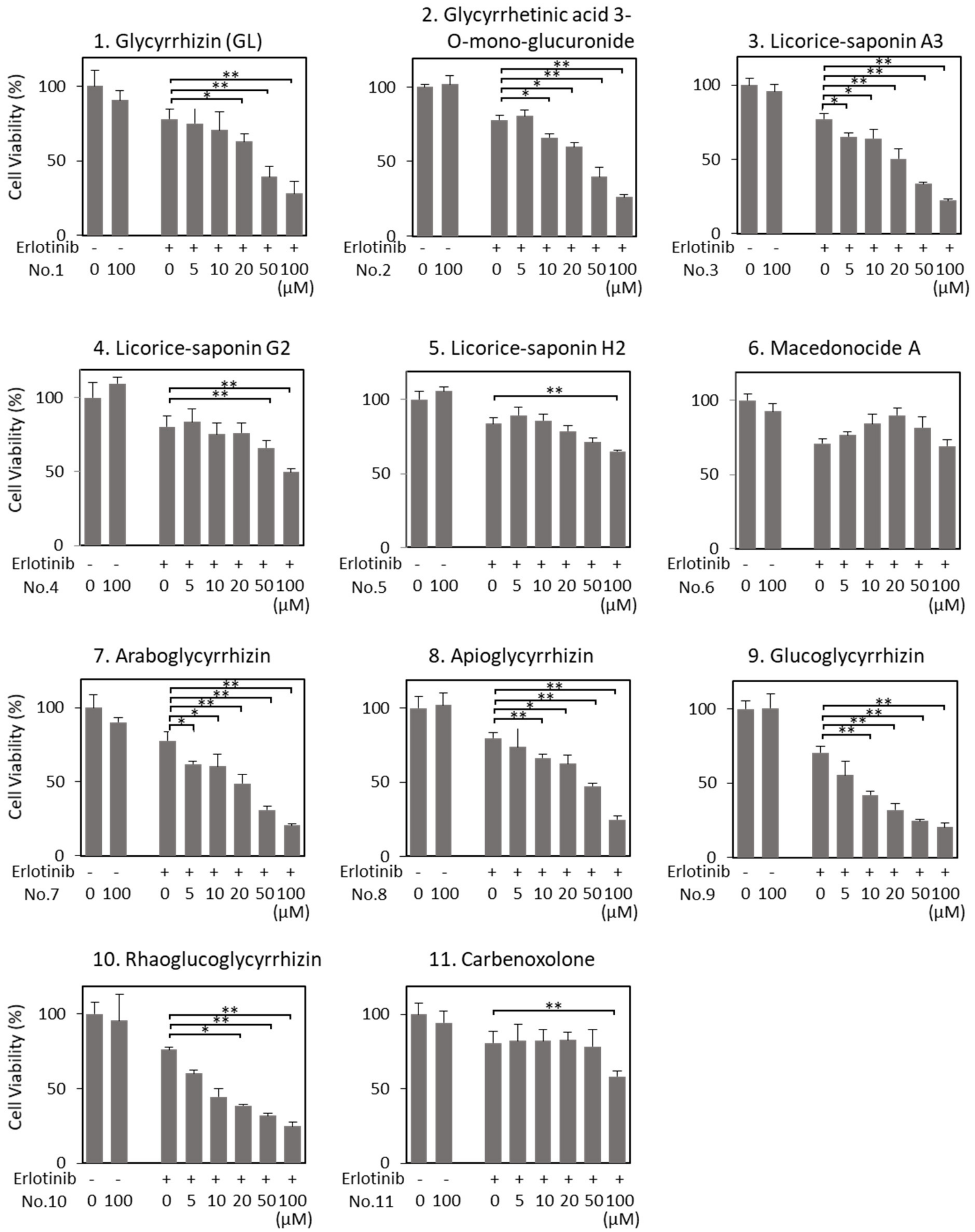

Figure S11. The effect of GL derivatives on erlotinib sensitivity in HCT116 cells. HCT116 cells were incubated with or without GL or GlucoGL treatment in the presence or absence of erlotinib for 12 h, and cell viability was examined using MTT assay. The data represent the mean ± SE of three separate experiments. * $p < 0.05$ or ** $p < 0.01$ using Student's *T* test.

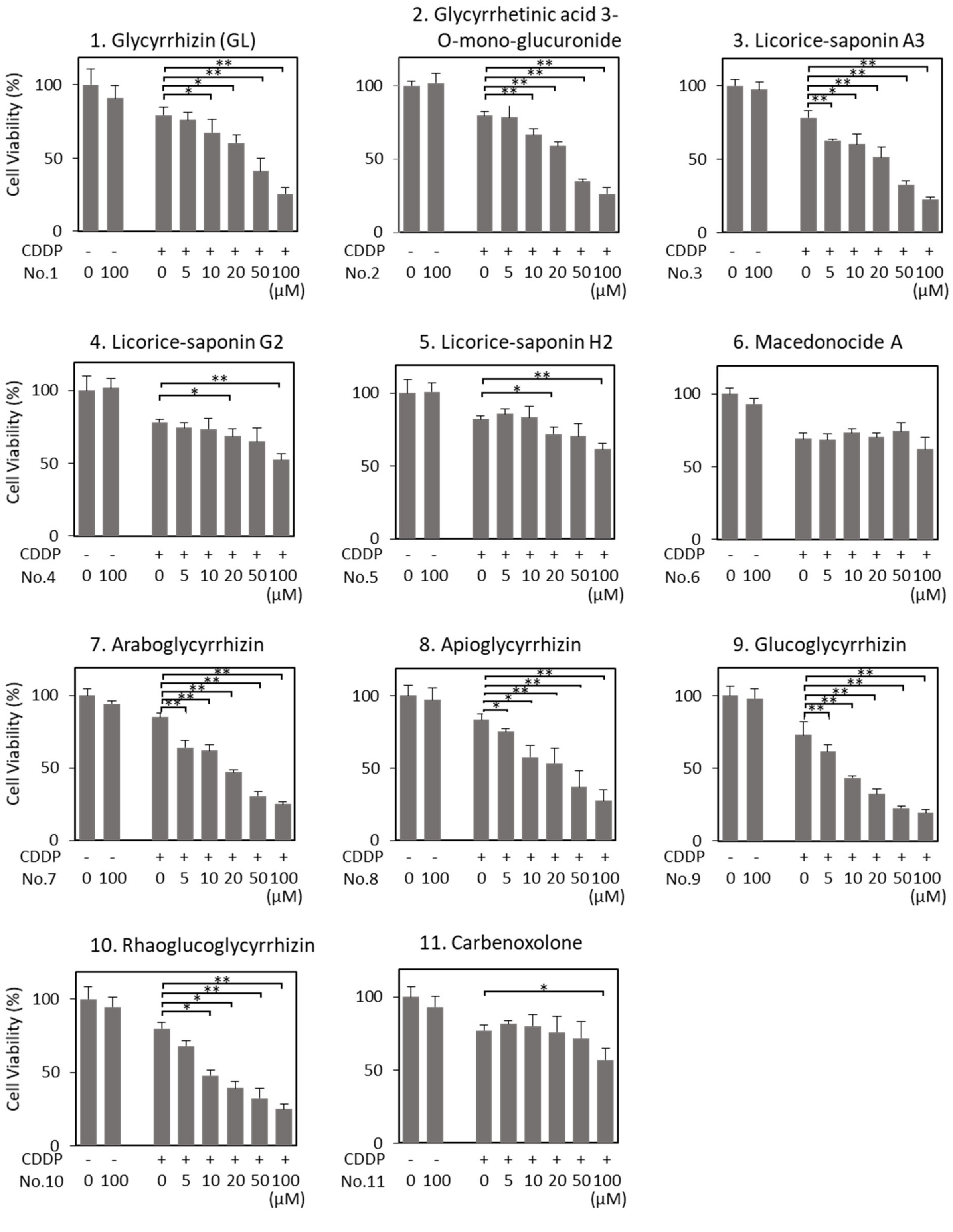

Figure S12. The effects of GL derivatives on CDDP sensitivity in HCT116 cells. HCT116 cells were incubated with or without GL or GlucoGL treatment in the presence or absence of CDDP for 12 h, and cell viability was examined using MTT assay. The data represent the mean ± SE of three separate experiments. * $p < 0.05$ or ** $p < 0.01$ using Student's *T* test.

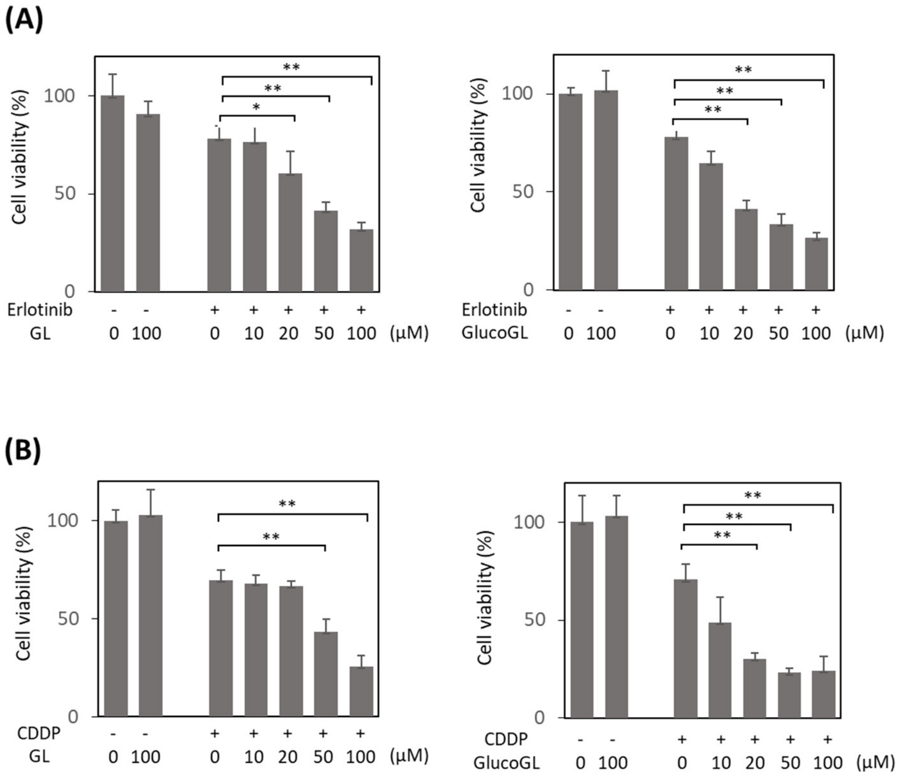

Figure S13. The effects of GL derivatives on erlotinib (A) or CDDP (B) sensitivity in HuH7 cells. HuH7 cells were incubated with or without GL or GlucoGL treatment in the presence or absence of CDDP for 12 h, and cell viability was examined using MTT assay. The data represent the mean ± SE of three separate experiments. * $p < 0.05$ or ** $p < 0.01$ using Student's *T* test.

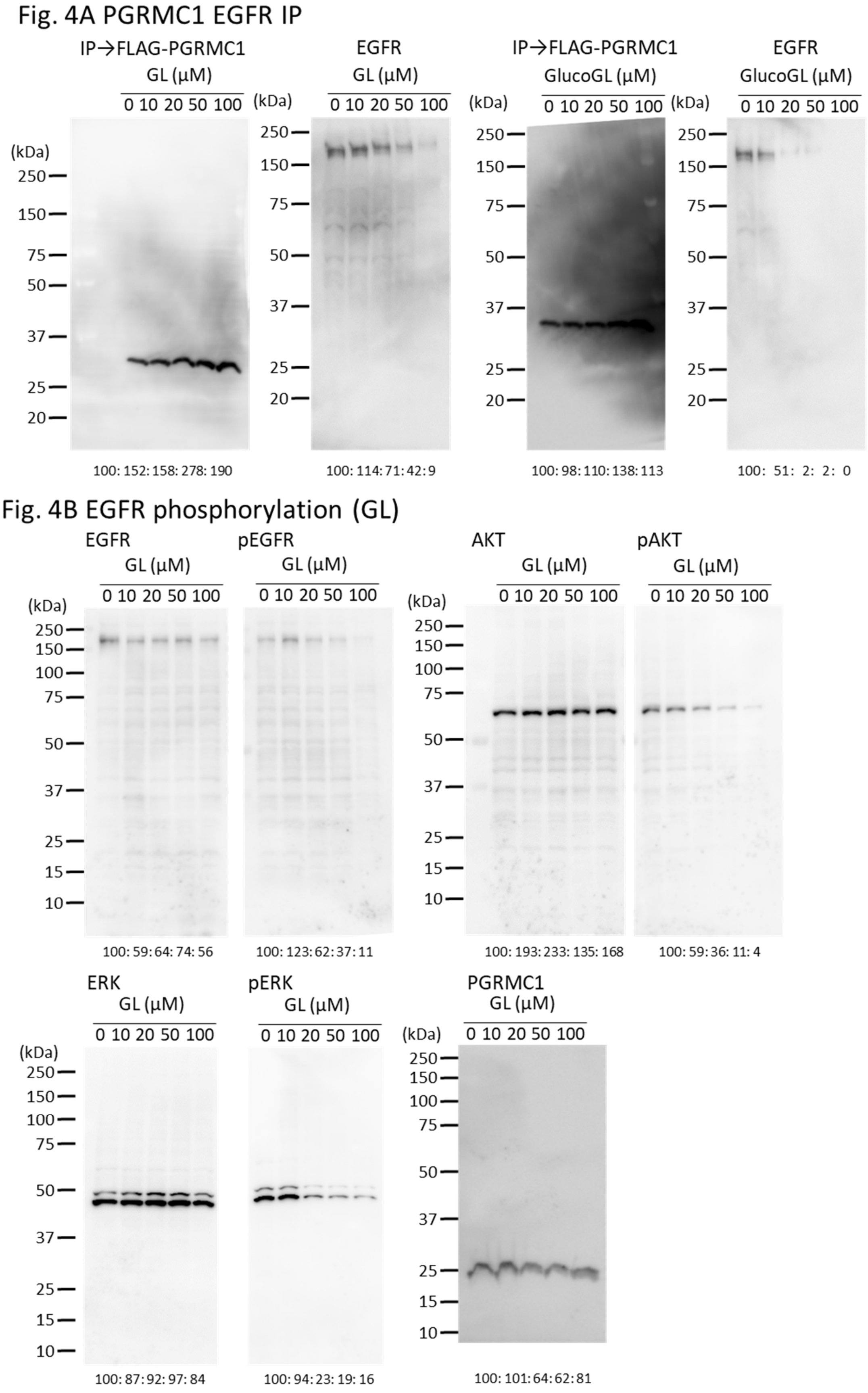
Fig. 4A PGRMC1 EGFR IP
IP→FLAG-PGRMC1
GL (μM)
0 10 20 50 100
EGFR
GL (μM)
0 10 20 50 100
IP→FLAG-PGRMC1
GlucoGL (μM)
0 10 20 50 100
EGFR
GlucoGL (μM)
0 10 20 50 100
(kDa)
250
150
75
50
37
25
20
100: 152: 158: 278: 190
100: 114: 71: 42: 9
100: 98: 110: 138: 113
100: 51: 2: 2: 0
Fig. 4B EGFR phosphorylation (GL)
EGFR
pEGFR
AKT
pAKT
GL (μM)
0 10 20 50 100
100
15
10
100: 59: 64: 74: 56
100: 123: 62: 37: 11
100: 193: 233: 135: 168
100: 59: 36: 11: 4
ERK
pERK
PGRMC1
100: 87: 92: 97: 84
100: 94: 23: 19: 16
100: 101: 64: 62: 81

Fig. 4B EGFR phosphorylation (GlucoGL)

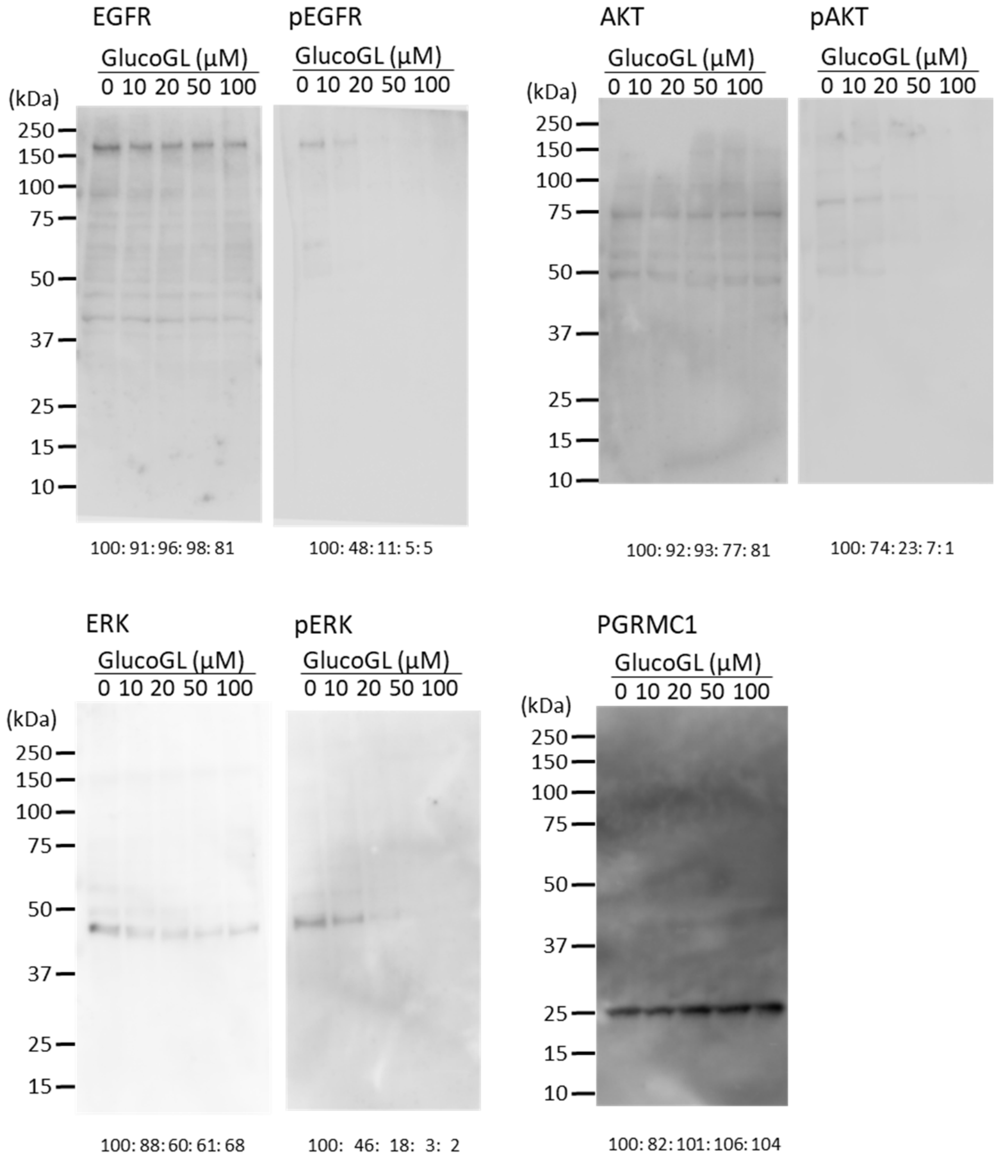

Fig. 5A PGRMC1 LDLR IP (HCT116)

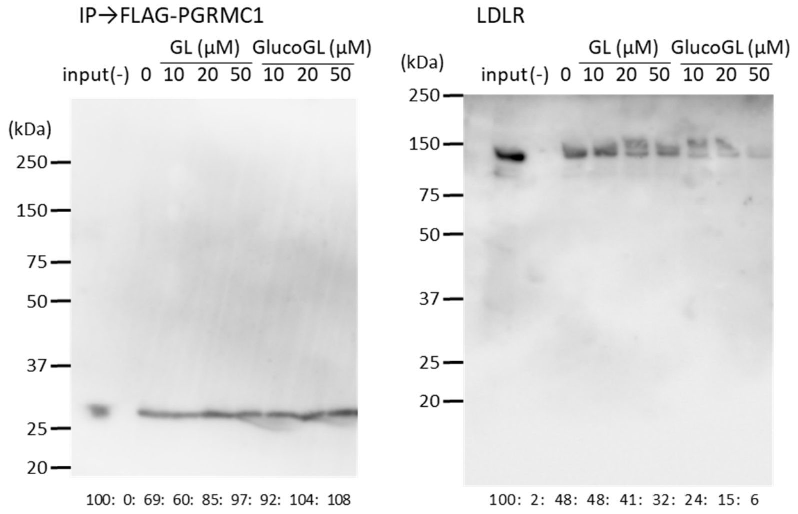

Fig. S6 FLAG PGRMC1 IP

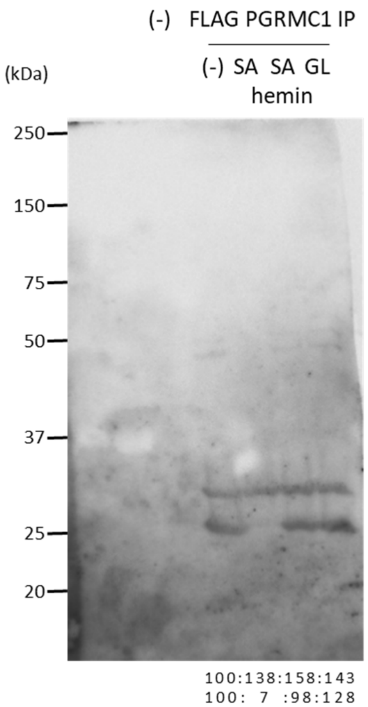

Fig. S7A PGRMC1 EGFR IP (HuH7)

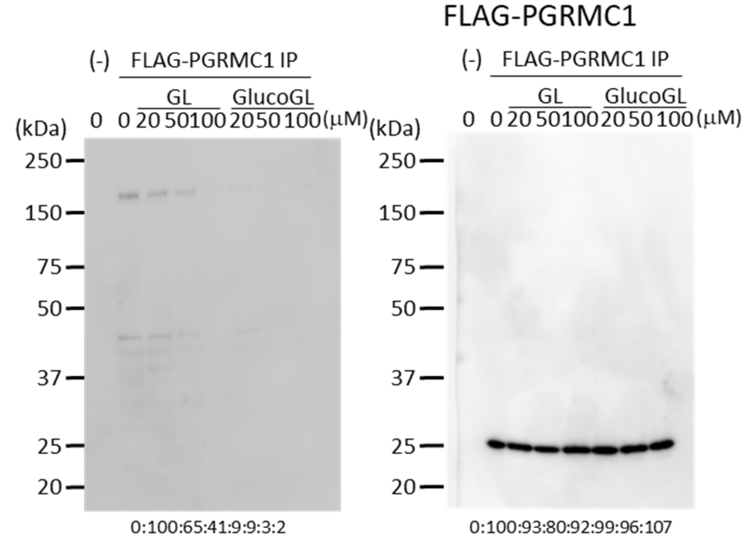

Fig. S7B EGFR phosphorylation (HuH7)

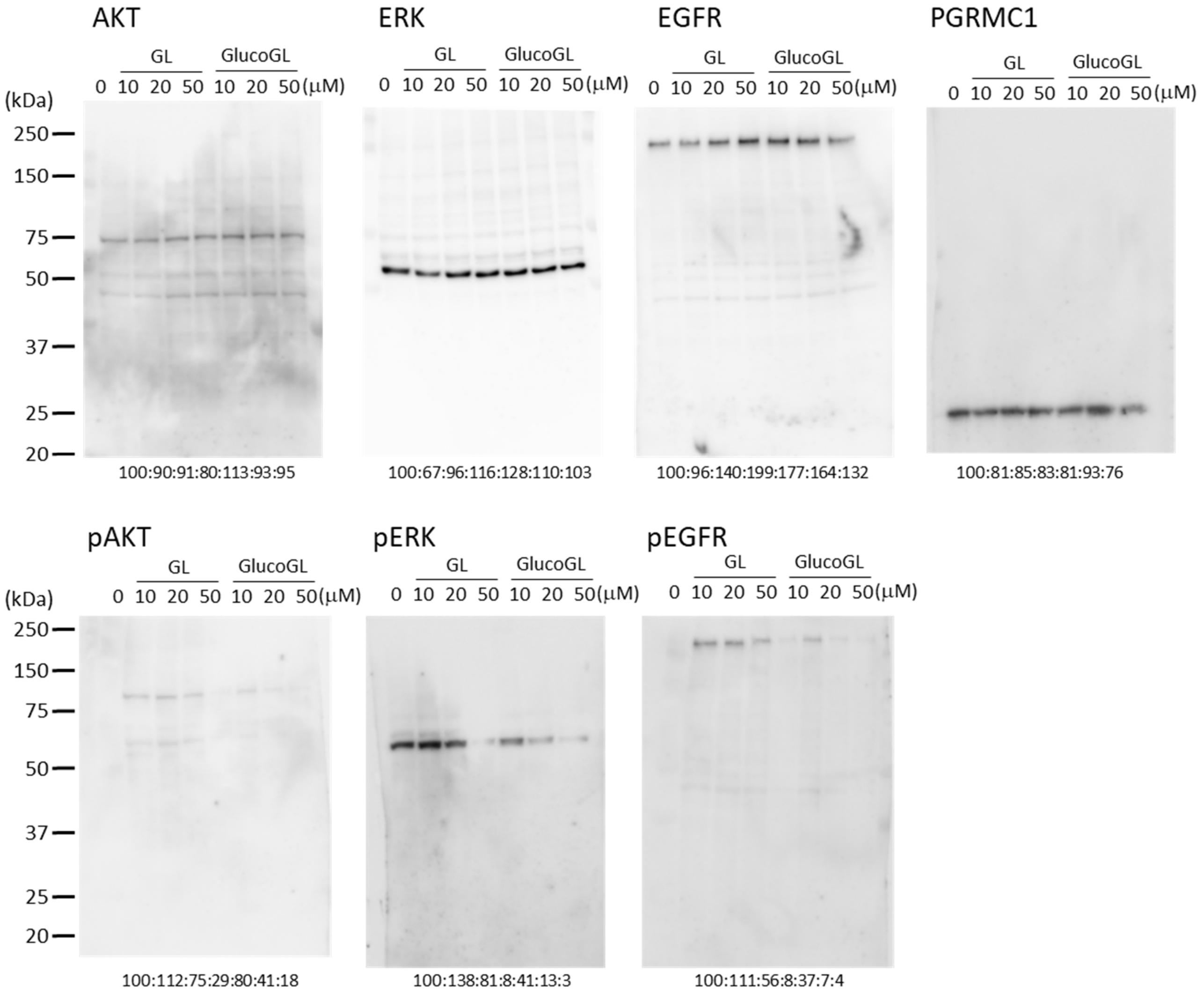

Fig. S8 EGFR phosphorylation (time course)

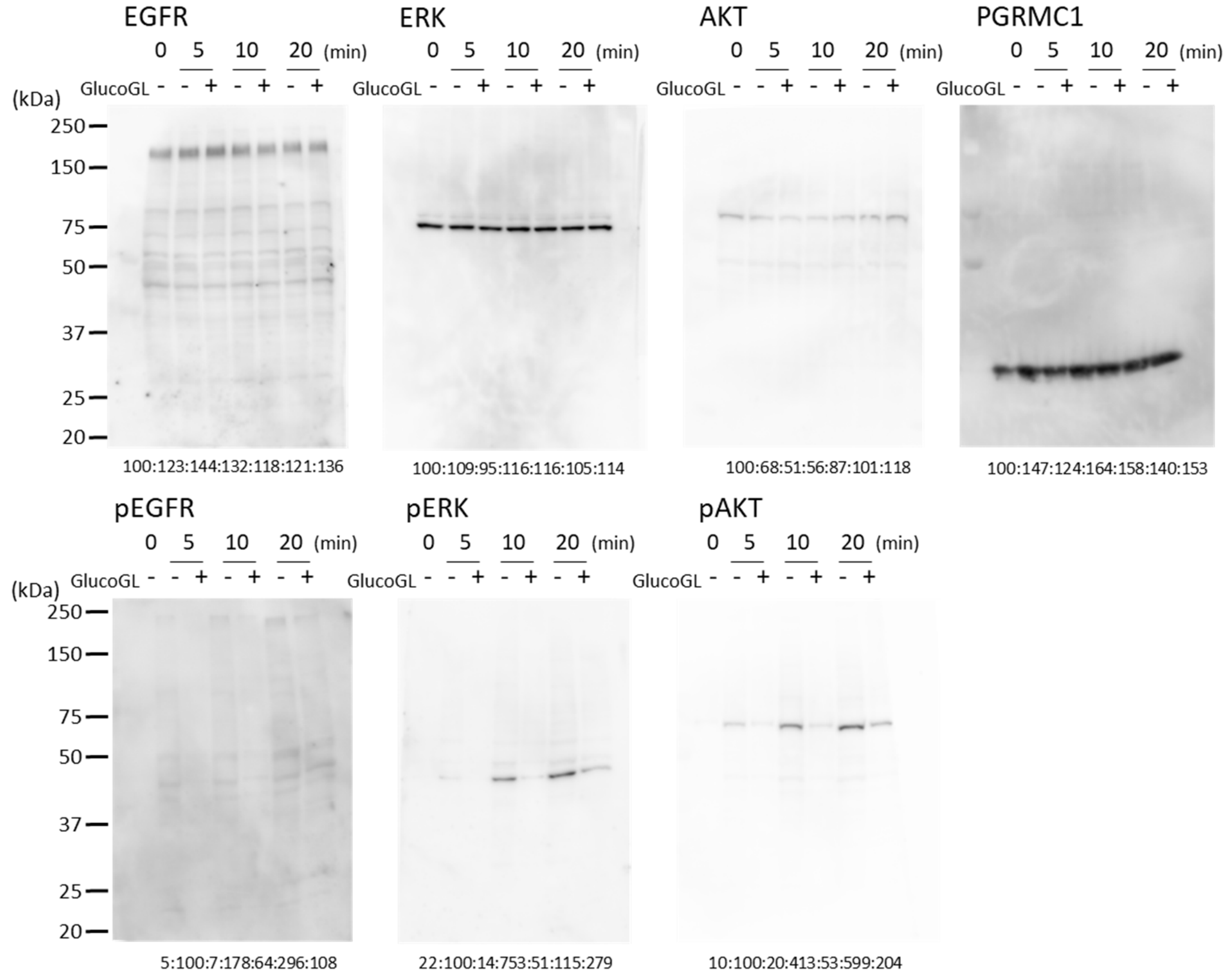

Fig. S10 PGRMC1 CYP3A4 IP

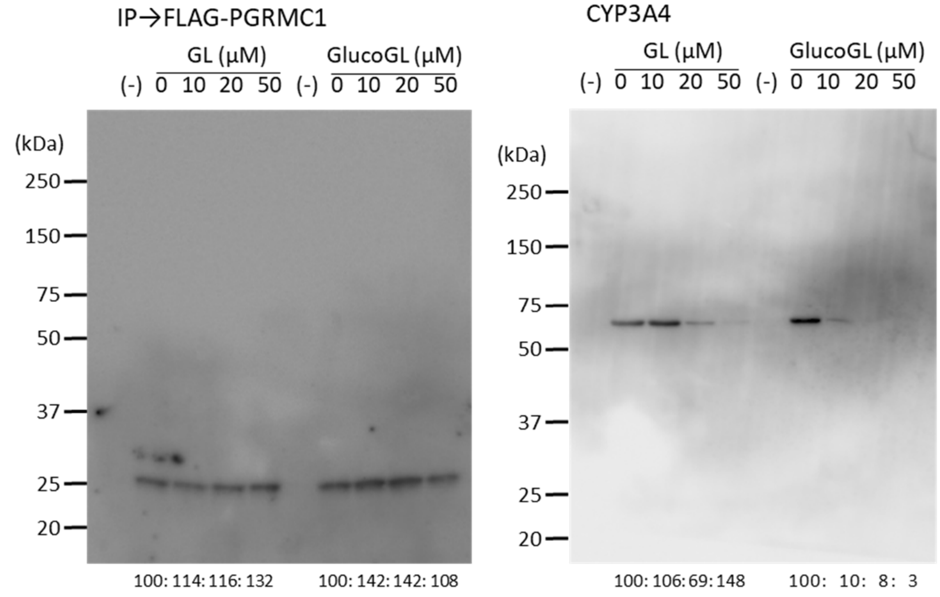

Figure S14. Full images of western blots.
